# Disruption of the rice *4-DEOXYOROBANCHOL HYDROXYLASE* unravels specific functions of canonical strigolactones

**DOI:** 10.1073/pnas.2306263120

**Published:** 2023-10-11

**Authors:** Guan-Ting Erica Chen, Jian You Wang, Cristina Votta, Justine Braguy, Muhammad Jamil, Gwendolyn K. Kirschner, Valentina Fiorilli, Lamis Berqdar, Aparna Balakrishna, Ikram Blilou, Luisa Lanfranco, Salim Al-Babili

**Affiliations:** ^a^The BioActives Lab, Center for Desert Agriculture, Biological and Environmental Science and Engineering Division, King Abdullah University of Science and Technology, Thuwal 23955-6900, Kingdom of Saudi Arabia; ^b^The Plant Science Program, Biological and Environmental Science and Engineering Division, King Abdullah University of Science and Technology, Thuwal 23955-6900, Kingdom of Saudi Arabia; ^c^Department of Life Sciences and Systems Biology, University of Torino, Torino 10125, Italy; ^d^Biological and Environmental Science and Engineering (BESE) Division, Plant Cell and Developmental Biology, King Abdullah University of Science and Technology, Thuwal 23955-6900, Saudi Arabia

**Keywords:** strigolactones, cytochrome P450, plant architecture, Striga, arbuscular mycorrhizal fungi

## Abstract

Strigolactones (SLs) regulate many developmental processes, including shoot-branching/tillering, and mediate rhizospheric interactions. SLs originate from carlactone (CL) and are structurally diverse, divided into a canonical and a noncanonical subfamily. Rice contains two canonical SLs, 4-deoxyorobanchol (4DO) and orobanchol (Oro), which are common in different plant species. The cytochrome P450 OsMAX1-900 forms 4DO from CL through repeated oxygenation and ring closure, while the homologous enzyme OsMAX1-1400 hydroxylates 4DO into Oro. To better understand the biological function of 4DO and Oro, we generated CRISPR/Cas9 mutants disrupted in *OsMAX1-1400* or in both *OsMAX1-900* and *OsMAX1-1400*. The loss of OsMAX1-1400 activity led to a complete lack of Oro and an accumulation of its precursor 4DO. Moreover, *Os1400* mutants showed shorter plant height, panicle and panicle base length, but no tillering phenotype. Hormone quantification and transcriptome analysis of *Os1400* mutants revealed elevated auxin levels and changes in the expression of auxin-related, as well as of SL biosynthetic genes. Interestingly, the *Os900/1400* double mutant lacking both Oro and 4DO did not show the observed *Os1400* architectural phenotypes, indicating their being a result of 4DO accumulation. Treatment of wild-type plants with 4DO confirmed this assumption. A comparison of the *Striga* seed germinating activity and the mycorrhization of *Os900, Os900/1400*, and *Os1400* loss-of-function mutants demonstrated that the germination activity positively correlates with 4DO content while disrupting *OsMAX1-1400* has a negative impact on mycorrhizal symbiosis. Taken together, our paper deciphers the biological function of canonical SLs in rice and reveals their particular contributions to establishing architecture and rhizospheric communications.

## Significance

Strigolactones (SLs) are multifunctional, structurally diverse secondary metabolites fulfilling the function of a hormone. Whether a particular SL exerts specific functions is one of the most important questions in SL biology. Here, we generated and characterized rice mutants lacking the common SLs 4-deoxyorobanchol and/or its derivative orobanchol, which represent one of the two SL subfamilies, i.e., canonical SLs. We show that 4-deoxyorobanchol is not a determinant of shoot branching, but has a specific function as a regulator of shoot, root, and panicle growth. Accumulation of 4-deoxyorobanchol affects auxin homeostasis and negatively impacts the symbiosis with mycorrhizal fungi. Our data reveal specific hormonal functions of canonical SLs and pave the way for targeted modulation of rice architecture and rhizospheric interactions.

Strigolactones (SLs) inhibit shoot branching and tillering (1, 2) and perform several regulatory functions related to stem thickness, leaf senescence, and stress responses (3, 4). They also promote the elongation of primary and seminal roots, but repress the formation of lateral and adventitious roots under insufficient phosphate (Pi) supply (5, 6). SLs were originally identified in root exudates as germination stimulants for root-parasitic weeds, such as *Orobanche* and *Striga* spp. (7), which pose severe problems for agriculture globally (8, 9). The reason why plants release SLs into the rhizosphere, particularly under Pi deficiency, was unraveled with the finding of their function as an initiation signal for establishing beneficial arbuscular mycorrhizal fungi (AMF) symbiosis, which induces hyphal branching in the fungal partner (10, 11).

SLs are derived from carotenoids and are characterized by a conserved butenolide ring (the D-ring; *SI Appendix*, Fig. S1) that is essential for their biological activity (12). The D-ring is linked via an enol-ether bridge to a second part that has a variable structure (3). Canonical SLs contain a tricyclic lactone (the ABC-ring) as the second moiety (*SI Appendix*, Fig. S1), whereas the noncanonical SLs have diverse and less defined structures (3, 13, 14). The core pathway in SL biosynthesis starts with a reversible isomerization of all-*trans*-β-carotene into 9-*cis*-β-carotene, catalyzed by DWARF27 (D27) (15). 9-*cis*-β-carotene is the substrate for the stereospecific carotenoid cleavage dioxygenase 7 (CCD7) that cleaves it into β-ionone and 9-*cis*-β-apo-10′-carotenal. In the next step, another CCD, CCD8, catalyzes a combination of reactions, including isomerization and repeated oxygenation, forming carlactone (CL), the core intermediate in SL biosynthesis, which is converted into different SLs in land plants (13, 16–20).

**Fig. 1.**
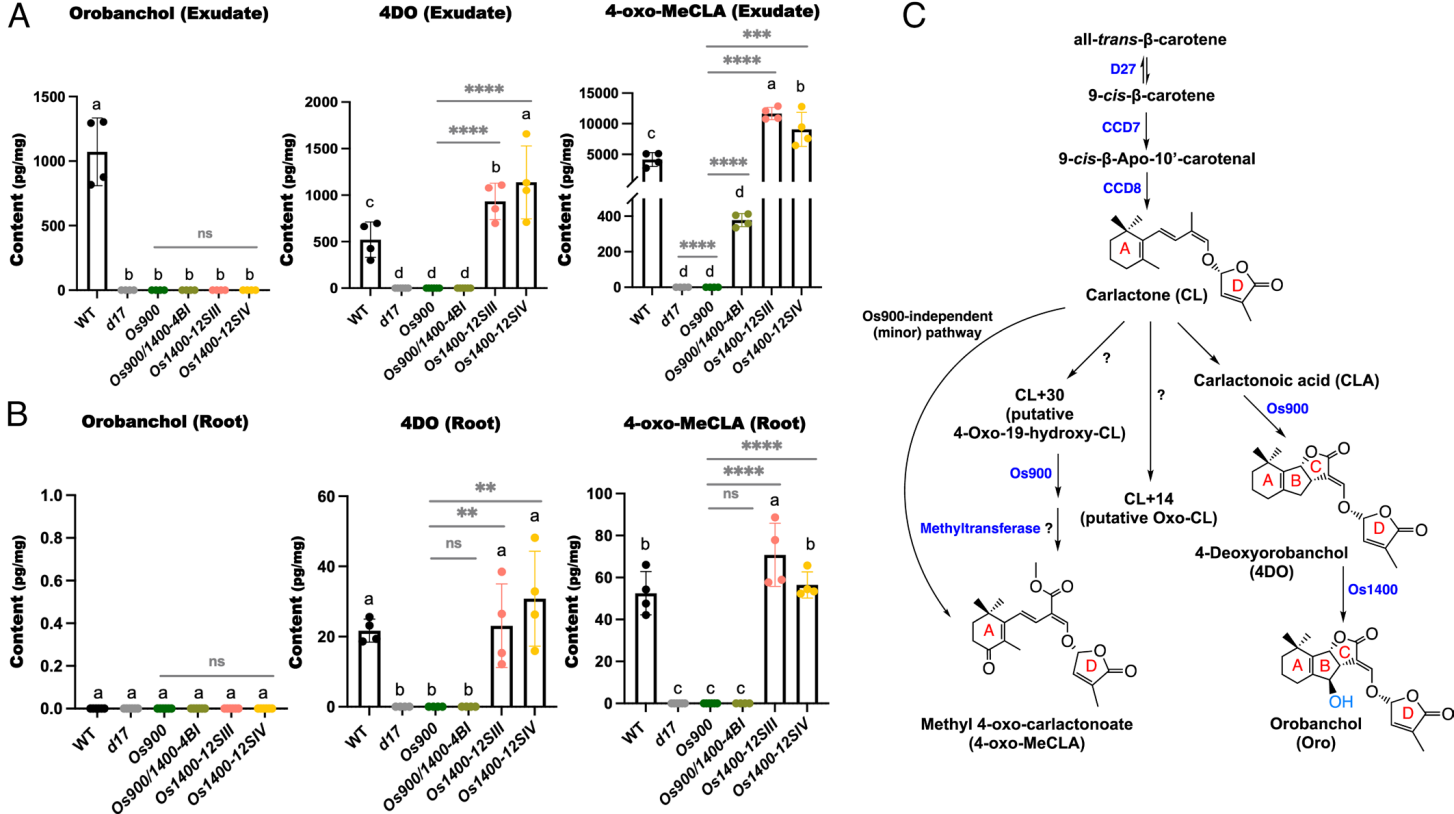
SL quantification of the CRISPR/Cas9 mediated *OsMAX1* mutants. (*A* and *B*) Analysis of SLs in root exudates and root tissues of WT*, Os900*-KO line*, Os900/1400*-KO line, *Os1400*-KO lines, and *d17* mutant grown under constant low-Pi conditions. 4-oxo-MeCLA is present in root exudates of WT and *Os900*-KO (at a quite low, less than 4% of the WT level; *SI Appendix*, Fig. S4*C*). The data are presented as means ± SD of 4 biological replicates for (*A*) and (*B*). Significant values determined by one-way ANOVA are shown with different letters (*P* < 0.05) when compared to WT, and asterisks indicate statistically significant differences as compared to control by the two tailed unpaired Student *t* test (*P < 0.05; **P < 0.01; ***P < 0.001; ****P < 0.0001). (*C*) Scheme of the rice SL biosynthesis. The pathway starts with a reversible isomerization of all-*trans*- into 9-*cis*-β-carotene, catalyzed by DWARF27 (D27). Next, 9-*cis*-β-carotene is transformed into carlactone (CL) through cleavage and rearrangement reactions mediated by the CAROTENOID CLEAVAGE DIOXYGENASE 7 and 8 (CCD7 and CCD8). OsMAX1 enzymes can oxygenate CL into carlactonoic acid (CLA), which is further converted into the canonical SLs 4-deoxyorobanchol (4DO) and then orobanchol (Oro) by sequential actions of Os900 and Os1400. In addition, CL is transformed by unknown enzymes into metabolites with a 14 Da (CL+14, putative Oxo-CL) and a 30 Da (putative 4-Oxo-19-hydroxy-CL) higher molecular mass. Os900 and a postulated methyltransferase convert CL+30 into the noncanonical SL methyl 4-oxo-carlactonoate (4-oxo-MeCLA, previously described as a methoxy-5-deoxystrigol isomer), which represents the major route for its formation. The detection of 4-oxo-MeCLA in exudates of *Os900-*KO mutant at a low level [not visible at the scale shown in (*A*), *SI Appendix*, Fig. S4*C*] indicates the presence of an additional, Os900-independent and minor route for its biosynthesis (21). Abbreviations: D27, Dwarf27; CCD, Carotenoid Cleavage Dioxygenase; MAX1, More Axillary Growth 1; CYP, Cytochrome P450; 4DO, 4-Deoxyorobanchol, 4-oxo-MeCLA, Methyl 4-oxo-carlactonoate.

More than 35 natural SLs have been identified in various plant species (12). For instance, several SLs have been found in rice, including the canonical SLs 4-deoxyorobanchol (4DO) and its hydroxylated derivative orobanchol (Oro), and the putative noncanonical SLs, CL+14, CL+30, and methyl 4-oxo-carlactonoate (4-oxo-MeCLA; previously described as a methoxy-5-deoxystrigol isomer) (21–23) (Fig. 1 and *SI Appendix*, Fig. S1). The structural diversity of SLs arises from the conversion of CL by different cytochrome P450 (CYP) and enzymes from other families, including MORE AXILLARY GROWTH1 (MAX1) from the CYP711A clade (23–25), the recently identified CYP722C, CYP712G1, CYP706C37 (26–28), and carlactonoic acid methyltransferase (12, 29).

The rice genome contains five homologs of Arabidopsis *MAX1*, namely Os01g0700900 (*OsMAX1-900*), *Os01g0701400* (*OsMAX1-1400*), *Os02g0221900* (*OsMAX1-1900*), *Os06g0565100* (*OsMAX1-5100*), and *Os01g0701500* (*OsMAX1-1500*, which encodes a truncated, nonfunctional enzyme in Nipponbare) (30–32). In vitro studies performed with yeast microsomes and transient expression in *Nicotiana benthamiana* have demonstrated that OsMAX1-900 mediates the conversion of CL into 4DO by repeated oxygenation and ring closure (21), whereas OsMAX1-1400 catalyzes the hydroxylation of 4DO into Oro (33). However, the enzymatic products of OsMAX1-1900 and OsMAX1-5100 remain unknown. These two enzymes do not convert CL into known SLs, indicating that they might be functionally distinct from other MAX1s (32, 34).

To understand the biological function(s) of specific SLs, we generated CRISPR/Cas9 rice mutant lines with the different *OsMAX1s*. Recently, we reported that *Osmax1-900* mutants do not show the most typical SL-deficiency phenotype, that is, increased tillering coupled with pronounced dwarfism, as in the SL-free *ccd7* (*d17*) mutant, albeit in the absence of 4DO and Oro (21). However, the lack of 4DO and Oro in *Osmax1-900* root exudates led to a significant decrease in the germination of *Striga* seeds, but caused a delay in AMF colonization. These findings suggest that the two canonical SLs, 4DO and Oro, are not the major tillering regulators in rice, which brings up the question whether they act as hormones in rice. In the present study, we aimed to address this question and identify the possible specific functions of 4DO and Oro in rice development and rhizospheric interactions. For this purpose, we generated *4-DEOXYOROBANCHOL HYDROXYLASE* (*Osmax1-1400*) single and *Osmax1-900/1400* double mutants and characterized them, using our previously described *Osmax1-900* (21) and *d17* (35) mutants as comparators.

## Results

### Disruption of *OsMAX1-1400* Leads to a Lack of Oro and Indicates Its Involvement in 4-oxo-MeCLA Conversion.

We generated two biallelic homozygous mutant lines with disrupted *OsMAX1-1400 (Os1400-12SIII* and *Os1400-12SIV,* *SI Appendix*, Fig. S2) and an *OsMAX1-900/1400 (Os900/1400-4BI*, *SI Appendix*, Fig. S3) double mutant line, using the CRISPR/Cas9 technology. Next, we determined and quantified the SLs in the roots and root exudates of these mutants under low-Pi conditions, using liquid chromatography-tandem mass spectrometry (LC-MS/MS) and compared them with those in *Osmax1-900 (Os900)* and SL-deficient *d17* mutants. As expected, the canonical SLs, 4DO and Oro, were not detected in *Os900* and *Os900/1400-4BI*, whereas *d17* did not contain both canonical and noncanonical SLs (Fig. 1 *A* and *B*, and *SI Appendix*, Fig. S4 *A* and *B*). The two *Os1400* mutants did not contain Oro, neither in the root tissue nor in exudates (Fig. 1 *A* and *B*). Interestingly, the *Os1400* mutants contained double amounts of 4DO and 4-oxo-MeCLA in root exudates compared with that in wild-type (WT) plants (Fig. 1*A*). Root exudates of the *Os900/1400-4BI* double mutant showed a lower level of 4-oxo-MeCLA, compared to that of both WT and *Os1400* mutants (Fig. 1*A*). The relative decrease in 4-oxo-MeCLA content was much more pronounced in the exudates of the *Os900* single mutant that released it in trace amounts (*SI Appendix*, Fig. S4*C*). We also quantified the noncanonical SLs, CL+30 and Oxo-CL (Fig. 1*C*) (21, 22), in the root exudates of *Os1400* mutants; however, we did not detect significant differences compared with that in WT exudates (*SI Appendix*, Fig. S4 *A* and *B*).

## Accumulation of 4DO in *Osmax1-1400* Mutants Has Different Effects On Shoot, Root, and Panicle Growth

**Fig. 2.**
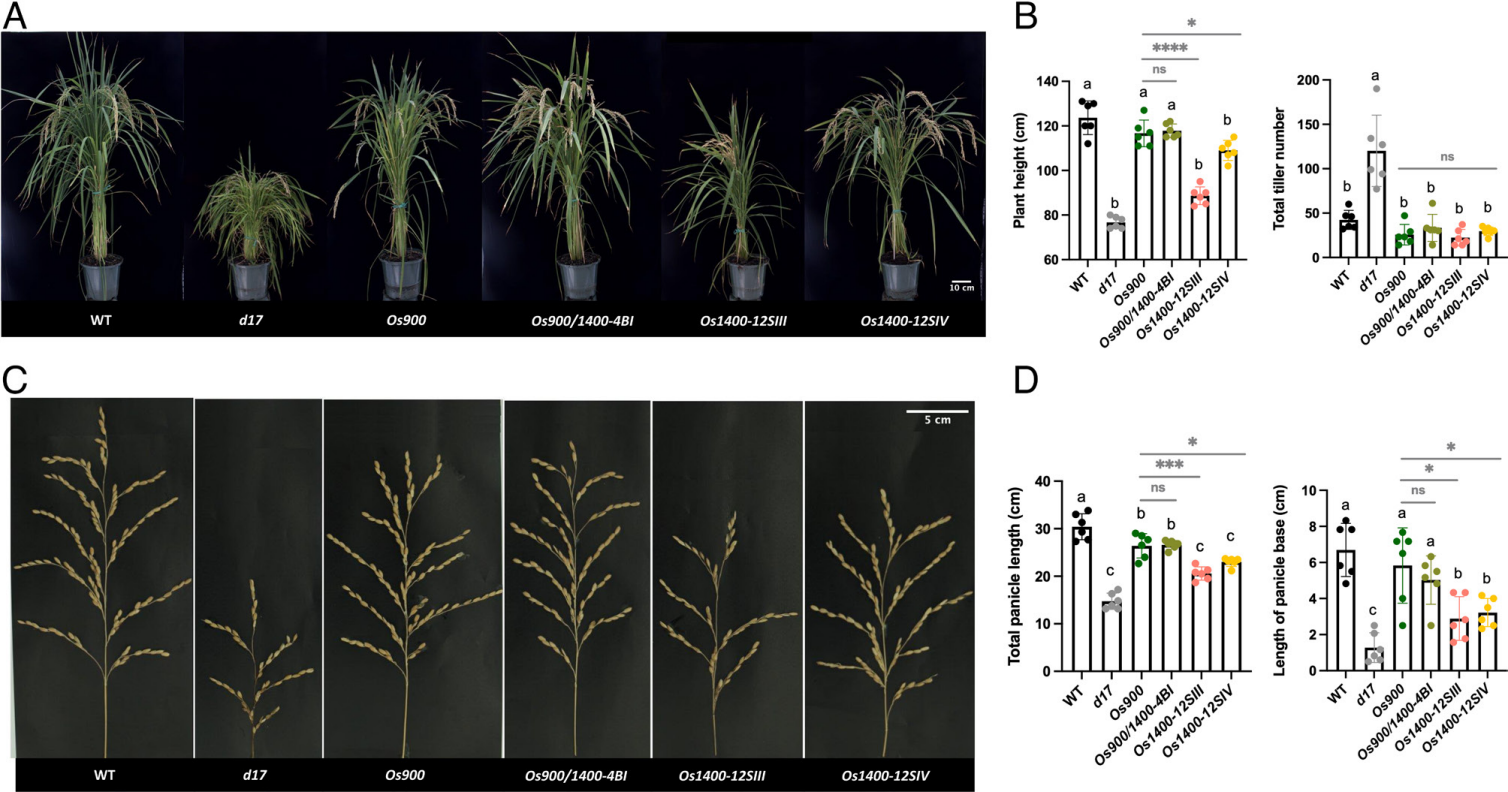
Phenotypic characterization of *OsMAX1* mutants. (*A* and *B*) Shoot phenotypes of WT, *Os900*-KO line, *Os900/1400-*KO line, *Os1400*-KO lines, and *d17* mutant grown in soil (Scale bar, 10 cm). (*C* and *D*) Panicle phenotypes of WT, *Os900*-KO line, *Os900/1400*-KO line, *Os1400*-KO lines, and *d17* mutant (Scale bar, 5 cm). The data are presented as means ± SD of six biological replicates. Significant values determined by one-way ANOVA are shown with different letter (*P* < 0.05) when compared to WT, and asterisks indicate statistically significant differences as compared to control by the two tailed unpaired Student *t* test (*P < 0.05; **P < 0.01; ***P < 0.001; ****P < 0.0001).

Next, we phenotyped *Os1400*, *Os900*, *Os900/1400-4BI, d17*, and WT plants in soil and hydroponic cultures. The *Os1400*, *Os900*, and *Os900/1400-4BI* mutants grown in the greenhouse did not show the typical SL-deficiency phenotypes, namely high tillering and dwarfism, observed in *d17* (Fig. 2*A*). However, plant height, panicle length, and panicle base length (the distance from the flag leaf auricle to the panicle base on the primary branch) in the *Os1400* mutants were significantly reduced, compared with those in the WT, *Os900*, and *Os900/1400* mutants (Fig. 2*B*). No significant differences were observed in the number of productive tillers, total number of tillers, and number of panicles under greenhouse conditions (*SI Appendix*, Fig. S5). A shorter shoot phenotype was also detected in the hydroponically grown *Os1400* mutants under both normal- and low-Pi conditions (*SI Appendix*, Figs. S6 and S8). Moreover, hydroponically grown *Os1400* mutants tended to have reduced length and diameter of roots under normal conditions (*SI Appendix*, Figs. S6 and S7), whereas an opposite phenotype, that is, a tendency for increased root length, under Pi deficiency, was observed compared with that in the WT plants (*SI Appendix*, Fig. S8). Interestingly, the *Os900/1400-4BI* double mutant, which lacks both 4DO and Oro, did not show the shoot, panicle, or root phenotypes observed in *Os1400* mutants (Fig. 2 *B* and *D* and *SI Appendix*, Figs. S6–S8), indicating that they were caused by the increased accumulation of 4DO rather than by the lack of Oro.

**Fig. 3.**
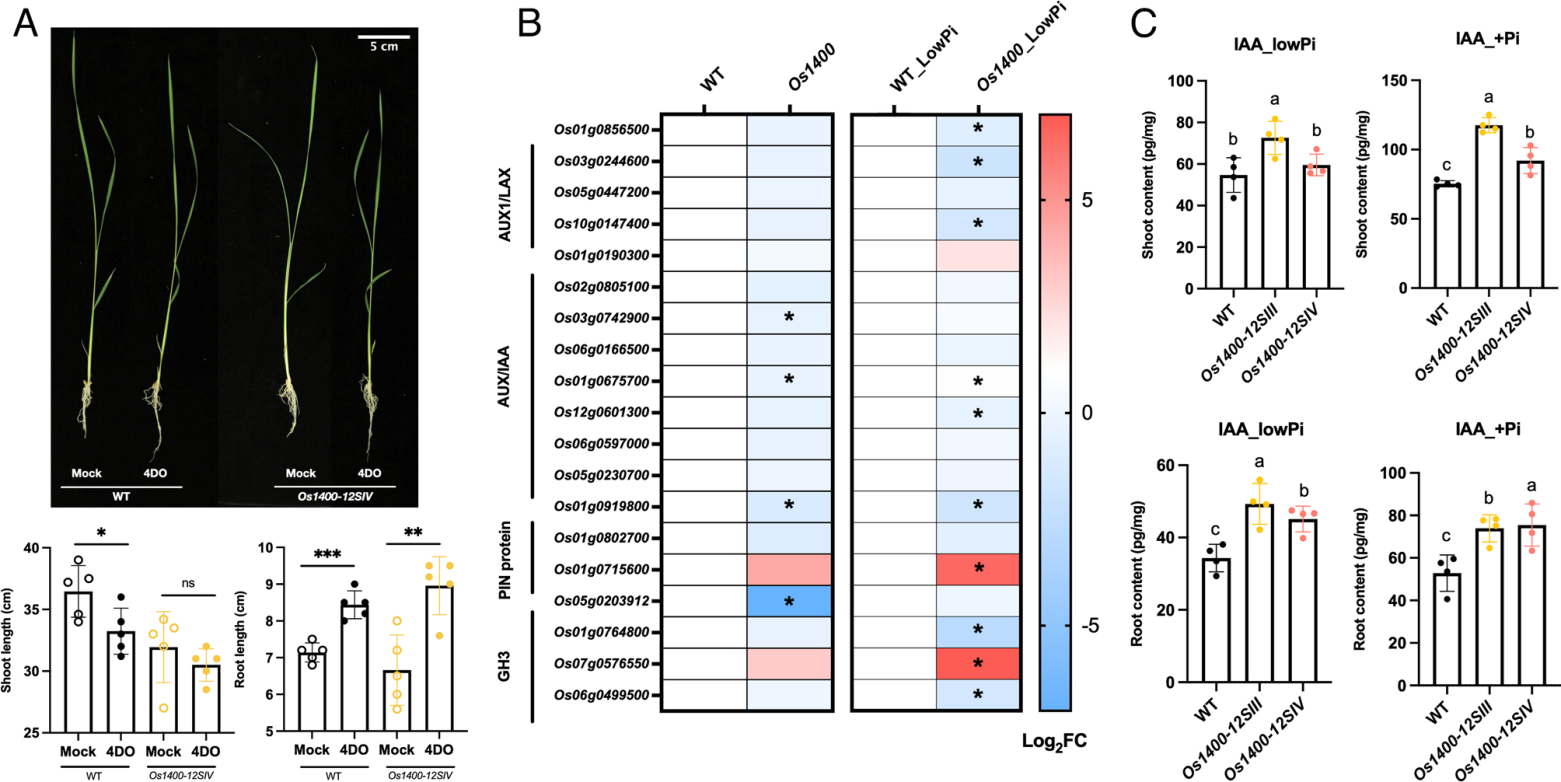
Exogenous 4DO application as well as transcriptome and hormone analysis of *Os1400*-KO lines. (*A*) Shoot and root phenotypes of WT, and *Os1400-*KO lines grown in hydroponic culture with or without (Mock) 900 nM 4DO (Scale bar, 5 cm). (*B*) Heat map analysis of DEGs involved Auxin pathways. AUXIN1/LIKE-AUX1 (AUX1/LAX) are major auxin influx carriers; AUXIN/indole-3-acetic acid (AUX/IAA) are transcriptional repressors; the PIN-FORMED (PIN) proteins are secondary transporters in the efflux of auxin; GRETCHEN HAGEN 3 (GH3) gene family encodes auxin-amido synthetases. The expression pattern was shown in log_2_FoldChange (Log_2_FC). Statistically significant differences are indicated by adjusted *P*-value (*< 0.05). (*C*) Analysis of IAA (auxin) in root and shoot of WT*, and Os1400*-KO lines grown under constant low-Pi and +Pi conditions. Abbreviation: 4DO, 4-deoxyorobanchol; WT, wild-type; ns, nonsignificant; IAA, indole-3-acetic acid. The data are presented as means ± SD of 5 (*A*), 3 (*B*), and 4 (*C*) biological replicates. Significant values determined by one-way ANOVA are shown with different letter (*P* < 0.05) when compared to WT, and asterisks indicate statistically significant differences as compared to control by the two tailed unpaired Student *t* test (*P < 0.05; **P < 0.01; ***P < 0.001; ****P < 0.0001).

To test this hypothesis, we treated the WT plants and *Os1400* mutants with 300 and 900 nM of 4DO under normal growth conditions. Application of 900 nM 4DO significantly reduced the shoot length in WT plants; however, *Os1400* mutants exhibited a tendency toward a further decrease in shoot length (Fig. 3*A*). Even at 300 nM, 4DO treatment led to a slight reduction in shoot length in the *Os1400* mutants (*SI Appendix*, Fig. S9). Moreover, at both concentrations under normal conditions, 4DO enhanced the root length in the *Os1400* and WT plants grown in hydroponics (Fig. 3*A* and *SI Appendix*, Fig. S9).

To further confirm the specific regulatory effects of 4DO, we treated *Os1400* seedlings grown under normal conditions with 5 µM TIS108, which inhibits OsMAX1-900 and OsMAX1-1400 (21), assuming that this treatment would restore the *Os1400* phenotypes, phenocopying the *Os900/1400-4BI* double mutant. As expected, TIS108 treatment significantly decreased the 4DO content in the root exudates of *Os1400* mutants, which was accompanied by accumulation of the noncanonical SLs, CL+30 and Oxo-CL (*SI Appendix*, Fig. S10). In addition, the root length and crown root number of the mutants treated with TIS108 were restored to those of the WT plants (*SI Appendix*, Fig. S11), indicating that the conversion of 4DO by OsMAX1-1400 is important for optimal root development in rice. Moreover, the shoot length in the *Os1400* mutants after TIS108 treatment was indistinguishable from that in the WT plants. However, we observed that application of TIS108 for 2 wk unexpectedly decreased the shoot length in the WT plants (*SI Appendix*, Fig. S11), probably due to the nonspecific effects of this cytochrome P450 inhibitor.

To gain deeper insights into the effect of *OsMAX1-1400* disruption, we compared the transcriptome of the *Os1400* mutants grown under normal or low-Pi conditions and the WT plants using RNAseq (Dataset S1). Among the differentially expressed genes (DEGs), we detected 1712 up-regulated and 1465 down-regulated genes under normal conditions, and 5890 up-regulated and 3569 down-regulated genes under low-Pi conditions (*SI Appendix*, Fig. S12). Under normal conditions, DEGs did not include any tillering- or SL biosynthesis-related genes (*SI Appendix*, Table S1), whereas under low-Pi conditions, a general downregulation of transcripts related to SL biosynthesis and signaling was noted (*SI Appendix*, Fig. S13 and Table S2). The decrease in the levels of these transcripts suggests that increased accumulation of 4DO in *Os1400* mutants triggers a negative feedback on the biosynthesis and perception of the SLs. It is speculated that this effect is not detectable under normal conditions because of the generally low SL content and expression of SL biosynthetic genes.

Interestingly, under both conditions, we observed a downregulation of many auxin-related genes, such as *GH3 auxin-amido synthetases*, in *Os1400* mutants (Fig. 3*B* and *SI Appendix*, Tables S1 and S2). Considering the essential role of auxins in determining the activity of meristems and in regulating shoot and root growth, as well as their impact on the levels of other hormones (36–38), we hypothesized that the phenotypes observed in the *Os1400* mutants may be linked to alterations in auxin content. Therefore, we determined the levels of auxin (IAA), gibberellin (GA), abscisic acid (ABA), salicylic acid (SA), and jasmonic acid (JA) in the roots and shoot bases (root-shoot junction) of *Os1400* mutants under normal and low-Pi conditions. We did not detect any significant difference in the levels of GAs, ABA, SA, or JA (*SI Appendix*, Fig. S14) between WT and the mutants; however, IAA showed a remarkable increase in the roots and shoot bases of the mutants under both growth conditions (Fig. 3*C* and *SI Appendix*, Fig. S14). Despite this, we did not observe clear differences in root meristem length between the *Os1400* mutants and WT plants (*SI Appendix*, Fig. S15).

### *Os1400* is Important for Rhizospheric Communications.

**Fig. 4.**
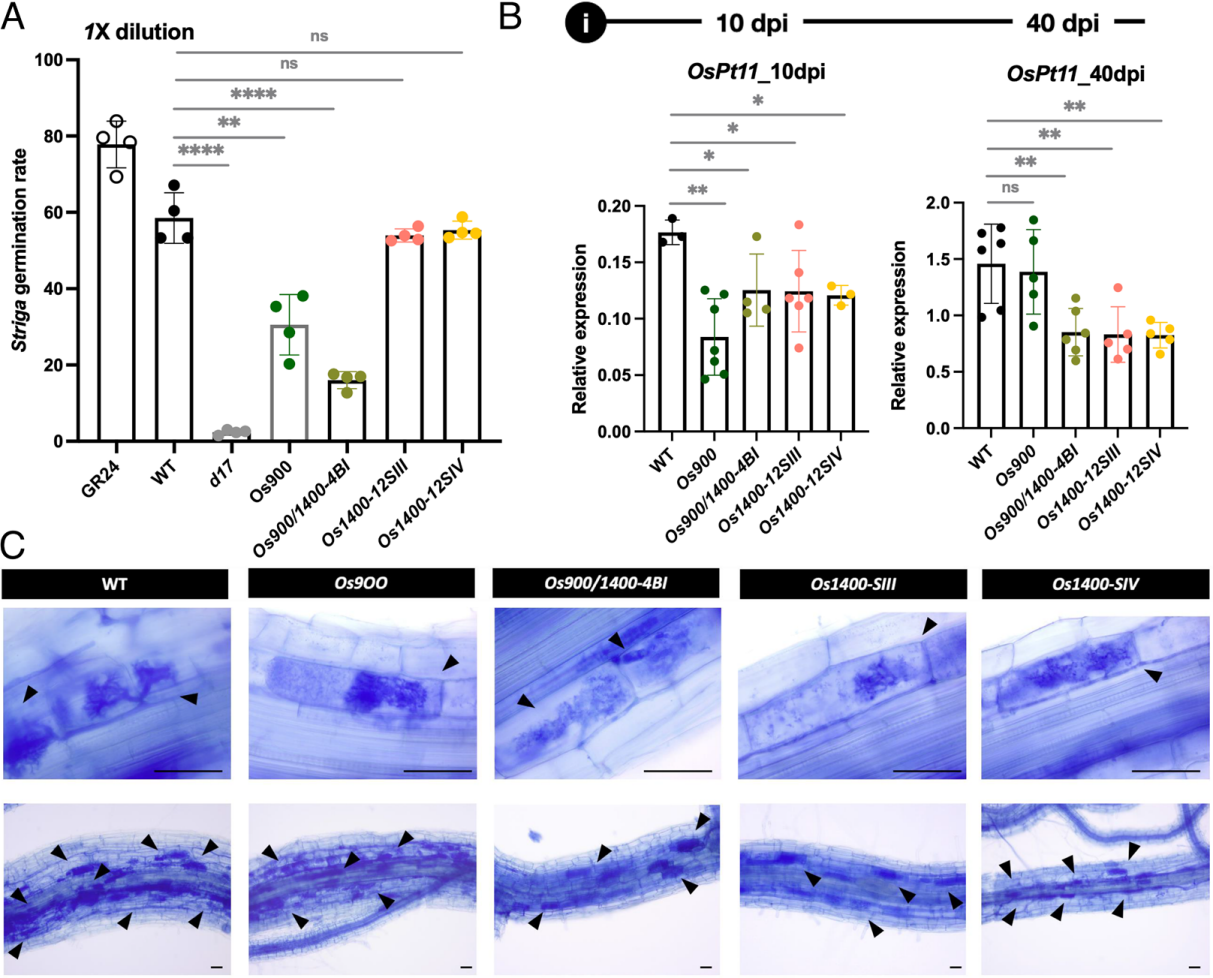
Assessment of rhizospheric interactions. Effect of *Os1400*-KO lines on (*A*) the germination of root parasitic weed *Striga* and (*B* and *C*) the arbuscule formation. The *R. irregularis* colonization was quantified by measuring the expression of an AM marker gene (*OsPT11*) (*B*). Arbuscule formation at 10 dpi and 40 dpi. Arrows indicate arbuscule containing cells (Scale bars, 50 µm). (*C*) The data are presented as means ± SD of 4 (*A*), and *n* ≥ 3 (*B*) biological replicates. Significant values determined by one-way ANOVA are shown with different letter (*P* < 0.05) when compared to WT, and asterisks indicate statistically significant differences as compared to control by the two tailed unpaired Student *t* test (*P < 0.05; **P < 0.01; ***P < 0.001; ****P < 0.0001).

We tested the effects of *Os900*, *Os1400*, and *Os900*/*1400* root exudates on the germination of *Striga hermonthica* seeds. The germination rate was decreased by more than 40% upon treatment with *Os900* exudates compared with that in WT exudate treatment, and the rate was further lowered with the *Os900/1400* exudate (Fig. 4*A*), indicating that 4-oxo-MeCLA, which is present at higher levels in the *Os900/1400* mutant exudates, is not a predominant germination signal for *Striga*. Although the effect of the *Os1400* exudate on the germination of *Striga* seeds was comparable to that of the WT at a 1:1 dilution, a tendency toward higher germination activity was observed when we diluted the exudates at a 1:3 ratio (Figs. 1*A* and 4*A*, and *SI Appendix*, Fig. S16 *A* and *B*). This result indicates that 4DO is a stronger germination cue for *Striga* seeds than Oro, as reported previously (39).

A previous study indicated that Oro is the preferred SL signal for inducing hyphal branching in AMF (40). To determine the effect of the lack of 4DO or Oro on AM symbiosis, we compared the mycorrhization of *Os900*, *Os1400, Os900*/1400, and WT plants. For this purpose, we examined root colonization with an AMF, *Rhizophagus irregularis*, at 10 and 40 d post inoculation (dpi) and measured the transcript levels of *OsPT11*, a specific AM-inducible Pi-transporter gene (41). At 10 dpi, the colonization of all mutant roots was delayed compared with that of WT roots, whereas at 40 dpi, the colonization of *Os1400* and *Os900*/*1400* mutants was surprisingly much lower than that of WT and *Os900* (Fig. 4*B* and *SI Appendix*, Fig. S17), indicating that *OsMAX1-1400* is crucial for maintaining optimal AM colonization. However, the absence of *OsMAX1-1400* did not influence the intraradical fungal structures of the arbuscules. Instead, they appeared well developed and regularly branched (Fig. 4*C*), suggesting that *OsMAX1-1400* did not affect the fungal morphology.

## Discussion

Recently, we showed that the lack of canonical SLs, 4DO and Oro, in *Os900* mutants does not significantly alter the shoot architecture, such as the high-tillering phenotype, which is observed in SL-deficient mutants, indicating that canonical SLs are not the major determinants of shoot branching in rice (21). These findings are in conformity with a recent report showing that the disruption of the two rice MAX1s, which likely mediate the biosynthesis of noncanonical SLs, leads to the expected high-tillering phenotype (32, 34). In contrast, the *Os900* mutant was affected in rhizospheric interactions and showed slightly modulated root growth, which indicates that canonical SLs have other biological functions (21). In the present study, we aimed to identify the roles of 4DO and Oro. For this purpose, we used the CRISPR/Cas9 technology to produce *Os1400* and *Os900/1400* loss-of-function mutants and compared them with the SL-free *d17* mutant and the *Os900* that lacks canonical SLs. We included the *Os900/1400* double mutant to account for the possibility that OsMAX1-1400 might utilize SLs produced by SL biosynthetic enzymes other than OsMAX1-900, and to ensure that *Os1400* phenotypes are solely a result of impaired conversion of 4DO into Oro. Analysis of SLs in the roots and root exudates of the *Os1400* mutants showed accumulation of 4DO accompanied by a lack of Oro, which confirms the function of OsMAX1-1400 as the 4DO hydroxylase in rice. Moreover, a comparison of the SL patterns in different mutant lines revealed an accumulation of 4-oxo-MeCLA in the root exudates of *Os900/1400* double mutant and *Os1400* single mutants (Fig. 1*A*). We also detected 4-oxo-MeCLA in *Os900* exudates, but at a low concentration (less than 4% of the WT level; *SI Appendix*, Fig. S4*C*), confirming the presence of a previously assumed Os900-independent route for the biosynthesis of this noncanonical SL (Fig. 1*C*) (21). This pattern indicates that OsMAX1-1400 may convert 4-oxo-MeCLA, a noncanonical SL likely formed by different SL biosynthetic enzymes including OsMAX1-900 (21), and points to a metabolic flux between OsMAX1-1400 and SL biosynthetic enzyme(s) other than OsMAX1-900. Furthermore, it can be concluded that OsMAX1-900 and OsMAX1-1400 may contribute to the synthesis of noncanonical SLs and that the canonical and noncanonical pathways interfere with each other. This may be due to the capability of OsMAX1s to produce different SLs and convert different substrates. This hypothesis could be supported by studies showing the conversion of CL into CLA by all OsMAX1 enzymes, including Os900 and Os1400, in in vitro and a heterologous plant system (33). Moreover, both canonical and noncanonical pathways share CL as a common precursor, which may indicate that blocking one of the two pathways increases the precursor availability for the other. Indeed, the increased accumulation of CL upon blocking the canonical pathway directed it to a metabolic shunt that led to the CL derivatives, CL+30 and Oxo-CL, in the *Os900* mutant (*SI Appendix*, Fig. S4 *A* and *B*), which may be the precursors of noncanonical SLs.

The *Os1400* mutants showed decreased shoot and panicle growth but no significant change in the tillering pattern, conforming to the notion of a negligible role of canonical SLs in inhibiting rice tillering (21) and pointing to other regulatory functions that affect root growth. These phenotypes indicate that the accumulation of 4DO had a negative effect on different growth-related processes in the *Os1400* mutants under normal Pi supply (*SI Appendix*, Fig. S6). However, under conditions of Pi deficiency, which further increased the 4DO content in the *Os1400* mutants, we observed an increase in root growth and a further decrease in shoot growth (*SI Appendix*, Fig. S8). These results indicate that 4DO generally has a negative effect on shoot growth, but its effect on root growth depends on Pi availability and its concentration. We conclude that 4DO promotes root growth under Pi deficiency, which usually triggers its biosynthesis, but acts as an inhibitor of root growth under normal conditions characterized by relatively low 4DO levels. Supporting our conclusion, neither the *Os900/1400* double mutant nor the *Os900* single mutant showed these shoot and root phenotypes, indicating that they were caused by 4DO accumulation rather than by a lack of Oro. It is speculated that the positive effect of exogenous application of 4DO on root growth under sufficient Pi supply (Fig. 3*A* and *SI Appendix*, Fig. S9) resembles the effect of increased 4DO production that occurs under Pi deficiency. Rice responds to insufficient Pi availability by increasing root elongation (42). To further confirm the importance of the conversion of 4DO into Oro for root growth and the effect of 4DO accumulation, we applied the inhibitor, TIS108, which blocks the activity of OsMAX1-900 and OsMAX1-1400, and hence, the formation of canonical SLs, to the roots of WT and mutant lines under normal Pi supply. Treatment with this chemical restored the root growth in the *Os1400* mutants (*SI Appendix*, Fig. S11). This result and the absence of *Os1400* growth phenotypes in the *Os900/1400* double mutant provide both genetic and chemical evidence for the importance of the OsMAX1-1400-dependent conversion of 4DO for normal growth and development of rice. Interestingly, a previous study showed that the deletion of *MAX1-900* and *MAX1-1400* was accompanied by a high-tillering phenotype in the Bala variety (23), which contradicts our results. However, it is speculated that the high-tillering phenotype of this variety is likely caused by other genetic factors, as we clearly show here that knocking out *MAX1-900* or *MAX1-1400*, or both, in the same genetic background, Nipponbare, did not increase the number of tillers (21, 22).

We observed a significant increase in auxin levels in the roots and shoot bases of the *Os1400* mutants, accompanied by the downregulation of several auxin-related transcripts, including genes encoding PIN-FORMED (PIN) proteins (Fig. 3 *B* and *C*). This increase may explain the observed decrease in shoot length, as the overexpression of the auxin transporter, *OsPIN2*, and exogenous IAA application have been shown to shorten shoots in rice (43, 44) and points to the role of 4DO as a positive regulator of auxin content. SLs also affect the transport and content of auxins in other plant species (45). For instance, Arabidopsis mutants lacking noncanonical SLs show elevated auxin content in shoot bases owing to increased PIN accumulation and enhanced auxin transport capacity (46, 47). Moreover, auxins and SLs are thought to affect the levels and distribution of each other to regulate axillary branching (45). It is speculated that increased 4DO levels interfere with the regulatory network connecting auxins with noncanonical SLs, mimicking a deficiency in these SLs, and thereby, having a positive effect on the content and transport of auxins. Further investigation is required to understand the interactions between different SL types and auxins in rice.

Depending on their structure, SLs differ in their efficiency to trigger seed germination in root-parasitic plants and in inducing hyphal branching in AMF (40). The different SL compositions of *Os900*, *Os900*/*1400*, and *Os1400* root exudates shown here (Fig. 1 *A* and *B*, and *SI Appendix*, Fig. S4) make them useful tools for characterizing the rhizospheric activity of specific SLs. Our results show that 4DO has a stronger ability than Oro to induce germination of *Striga* seeds. Recently, we showed that the lack of both Oro and 4DO in *Os900* only caused a delay in mycorrhization. One would expect that the *Os900/1400* double mutant showed the same delay in mycorrhization; however, we observed a steadily lower mycorrhization rate in the double mutant as well as in *Os1400* single mutants, indicating an important role of OsMAX1-1400 in mycorrhization, which might be independent of its function in Oro formation.

Taken together, we confirm that canonical SLs do not exert the best-known hormonal function of SLs, i.e., inhibition of tillering, but play a role in other developmental processes and are important for proper rhizospheric communication. In particular, our data demonstrate that 4DO has a pleiotropic effect on the growth of the root, shoot, and panicle and that its accumulation modulates auxin homeostasis. Moreover, we can conclude that the rice canonical SLs, 4DO and Oro, are important for the interaction with AMF and root-parasitic plants. Blocking the biosynthesis of both 4DO and Oro does not significantly affect rice architecture but can significantly alleviate infestation by root-parasitic plants, such as *Striga*, offering a strategy for managing weeds that threaten global food security.

## Materials and Methods

### Plant Material and Growth Conditions.

*Oryza sativa* Nipponbare *d17* (35), *max1-900* (21), *max1-1400*, *max1-900/1400*, and WT rice plants were grown under controlled conditions (a 12 h photoperiod, 200-µmol photons m^−2^ s^−1^ and day/night temperature of 27/25 °C). All rice seeds were first surface-sterilized in a 50% commercial bleach (sodium hypochlorite) solution (Milli-Q water + commercial bleach; 1:1 v/v) with 0.01 % Tween-20 for 15 min, then rinsed with sterile water, before being germinated in the dark overnight. The pregerminated seeds were placed on Petri dishes containing half-strength liquid Murashige and Skoog (MS) medium and incubated in a growth chamber for 7 d. Thereafter, the seedlings were transferred into 50 mL black falcon tubes filled with half-strength modified Hoagland nutrient solution with adjusted pH to 5.8. The nutrient solution consisted of 5.6 mM NH_4_NO_3_, 0.8 mM MgSO_4_·7H_2_O, 0.8 mM K_2_SO_4_, 0.18 mM FeSO_4_·7H_2_O, 0.18 mM Na_2_EDTA·2H_2_O, 1.6 mM CaCl_2_·2H_2_O, 0.8 mM KNO_3_, 0.023 mM H_3_BO_3_, 0.0045 mM MnCl_2_·4H_2_O, 0.0003 mM CuSO_4_·5H_2_O, 0.0015 mM ZnCl_2_, 0.0001 mM Na_2_MoO_4_·2H_2_O, and 0.4 mM K_2_HPO_4_·2H_2_O.

### Generation of *Os1400* and *Os900/1400* Plants.

Two guide RNAs [gRNAs; single gRNA3 (sgRNA3), 5′ -tgcgaacaggttgaaattgg-3′ and sgRNA4, 5′ -ctcgagtttcagtactcgat-3′] were designed to target the rice (*O. sativa* L. ssp. japonica cv. Nipponbare) *OsMAX1-1400* (Os01g0701400 /AP014957) gene. By using Golden Gate cloning, the tRNA-gRNA-Cas9 cassette was assembled into the pRGEB32 binary vector that has hygromycin resistance gene for selection. With mature seeds, Nipponbare calli were induced and transformed with *Agrobacterium tumefaciens* EHA105 culture containing the plasmid of interest. Later, shoots and roots were regenerated in a Percival growth chamber (CLF Plant Climatics GmbH, model CU 36L5), and then transferred to soil and grown in a greenhouse at 28 °C day/22 °C night.

Genomic DNA was extracted from young leaves, and plant transgenicity and mutagenicity were demonstrated. Using polymerase chain reaction (PCR) amplification, the transgenic plants were recognized when the pRGEB32-specific primers, pRGEB32-F (5′-ccacgtgatgtgaagaagtaagataaactg-3′) and pRGEB32-R (5′- gataggtttaagggtgatccaaattgagac-3′), bind to the surrounding region of the insertion sites in the pRGEB32 vector. For identifying CRISPR-mediated mutations, the DNA region that includes the sgRNA target sites was amplified using genome specific primers *Os1400*-sg3-sg4-F (5′-tcagcgcgctcacttacga-3′), *Os1400*-sg4-F1 (5′-atcccaagaacttcccggag-3′), *Os900*-sg3-sg4-F (5′-gccatactggaaagtgcgg-3′), *Os900-*sg3-sg4-R (5′-tagcttcaggtaaaattgcgcg-3′), *Os1400*-sg1-sg2-F3 (5′-atgcaggggttcgaggtg-3′), and *Os1400*-sg1-sg2-R3 (5′-gttggccgatgatgatggac-3′) (*SI Appendix*, Table S3).

### Hydroponic Culture of Rice Seedlings.

The hydroponic culture system is built with 50-mL black falcon tubes with punctured caps inserted with a 1.5-mL bottomless Eppendorf tube in the center. Nutrition solution, containing normal (+Pi) or low 0.004 mM K_2_HPO_3_·3H_2_O (low-Pi), was applied to the transferred 1-wk-old seedlings for the following 2 wk. The solutions were changed every 3 d, and adjusted to pH 5.8 every time before applying. All plants were kept in the solution for 3 wk, except the plants for 4DO application and EdU staining were 10-d seedlings.

### Phenotyping in Pots Under Greenhouse Conditions.

To study the phenotype of *Os1400* and *Os900/1400* mutants, seedlings were transferred into pots packed with soil. The soil was soaked with half-strength modified Hoagland nutrient solution in advance. The nutrient solution consisted of 5.6 mM NH_4_NO_3_, 0.8 mM MgSO_4_.7H_2_O, 0.8 mM K_2_SO4, 0.18 mM FeSO_4_.7H_2_O, 0.18 mM Na_2_EDTA.2H_2_O, 1.6 mM CaCl_2_.2H_2_O, 0.8 mM KNO_3_, 0.023 mM H_3_BO_3_, 0.0045 mM MnCl_2_.4H_2_O, 0.0003 mM CuSO_4_.5H_2_O, 0.0015 mM ZnCl_2_, 0.0001 mM Na_2_MoO_4_.2H_2_O, and 0.4 mM K_2_HPO_4_.2H_2_O. The pH of the solution was adjusted to 5.8, and the solution was applied every third day. On day 120, phenotypic data were recorded. The plants were grown in a greenhouse from February to May 2022 in Thuwal (Saudi Arabia).

### Exogenous Applications of 4DO and TIS108.

For investigating the effect of 4DO (Olchemim, Czech Republic) on different genotypes, 1-wk-old seedlings were grown hydroponically in half-strength Hoagland nutrient solution containing 0.4 mM K_2_HPO_4_·2H_2_O (+Pi), 300 nM or 900 nM 4DO (dissolved in acetone), or the corresponding volume of the solvent (mock; acetone) for 10 or 14 d. The solution was changed three times per week, adding the chemical at each renewal.

For investigating the effect of TIS108, 2-wk-old rice seedlings were grown hydroponically in half-strength Hoagland nutrient solution containing 0.4 mM K_2_HPO_4_·2H_2_O (+Pi), 5 µM TIS108 (dissolved in acetone), or the corresponding volume of the solvent (mock; acetone) for 14 d. The solution was changed twice per week, adding the chemical at each renewal.

### SL Quantification in Root Tissues and Exudates.

Analysis of SLs in rice root exudates was performed according to the published protocol (48). Briefly, root exudates spiked with 2 ng of GR24 were brought on a C_18_-Fast Reversed-SPE column (500 mg/3 mL), preconditioned with 3 mL of methanol and followed with 3 mL of water. After washing with 3 mL of water, SLs were eluted with 5 mL of acetone. Thereafter, SLs-containing fraction was concentrated to SL aqueous solution (∼500 μL), followed by 1 mL of ethyl acetate extraction. 750 μL of SL enriched fraction was dried under vacuum. The final extract was redissolved in 100 μL of acetonitrile: water (25:75, v:v) and filtered through a 0.22-μm filter for LC-MS/MS analysis.

SL extraction from root tissues was performed following the procedure by Wang et al. (49). Around 25 mg of lyophilized and grinded rice root tissues, spiked with 2 ng of GR24, were extracted twice with 2 mL of ethyl acetate in an ultrasound bath (Branson 3510 ultrasonic bath) for 15 min, followed by centrifugation for 8 min at 3,800 rpm at 4 °C. The two supernatants were combined and dried under vacuum. The residue was dissolved in 50 μL of ethyl acetate and 2 mL of normal hexane and applied to a Silica Cartridges SPE column (500 mg/3 mL). After washing with 3 mL of hexane, SLs were eluted in 3 mL of ethyl acetate and evaporated to dryness under vacuum. The final extract was redissolved in 150 μL of acetonitrile: water (25:75, v:v) and filtered through a 0.22 μm filter for LC-MS/MS analysis.

SLs were quantified by LC–MS/MS using the UHPLC-Triple-Stage Quadrupole Mass Spectrometer (Thermo Scientific^TM^ Altis^TM^). Chromatographic separation was achieved on the Hypersil GOLD C_18_ Selectivity HPLC Columns (150 × 4.6 mm; 3 μm; Thermo Scientific^TM^) with mobile phases consisting of water (A) and acetonitrile (B), both containing 0.1% formic acid, and the following linear gradient (flow rate, 0.5 mL/min): 0–15 min, 25 %–100 % B, followed by washing with 100 % B and equilibration with 25 % B for 3 min. The injection volume was 10 μL, and the column temperature was maintained at 35 °C for each run. The MS parameters of Thermo ScientificTM Altis^TM^ were as follows: positive ion mode, ion source of H-ESI, ion spray voltage of 5,000 V, sheath gas of 40 arbitrary units, aux gas of 15 arbitrary units, sweep gas of 20 arbitrary units, ion transfer tube gas temperature of 350 °C, vaporizer temperature of 350 °C, collision energy of 17 eV, CID gas of 2 mTorr, and full width at half maximum (FWHM) 0.2 Da of Q1/Q3 mass. The characteristic Multiple Reaction Monitoring (MRM) transitions (precursor ion → product ion) were 331.15 → 216.0, 331.15 → 234.1, 331.15 → 97.02 for 4-deoxyorobanchol; 347.14 → 329.14, 347.14 → 233.12, 347.14 → 205.12, 347.14 → 97.02 for orobanchol; 361.16 → 247.12, 361.16 → 177.05, 361.16 → 208.07, 361.16 → 97.02 for putative 4-oxo-MeCLA; 333.17 → 219.2, 333.17 → 173.2, 333.17 → 201.2, 333.17 → 97.02 for putative 4-Oxo-hydroxyl-CL (CL+30); 317.17 → 164.08, 317.17 → 97.02 for putative Oxo-CL (CL+14); 299.09 → 185.06, 299.09 → 157.06, 299.09 → 97.02 for GR24.

### Quantification of Plant Hormones.

Quantification of endogenous hormones was performed following the procedure of (50). Briefly, 15 mg freeze-dried grinded root or shoot base tissues were spiked with internal standards D6-ABA (10 ng), D2-GA1 (10 ng), D2-IAA (10 ng), D4-SA (10 ng), and D2-JA (10 ng) along with 750 µL of methanol. The mixture was sonicated for 15 min in an ultrasonic bath (Branson 3510 ultrasonic bath), followed by centrifugation for 5 min at 14,000×*g* at 4 °C. The supernatant was collected, and the pellet was re-extracted with 750 µL of the same solvent. Then, the two supernatants were combined and dried under vacuum. The sample was redissolved in 100 μL of acetonitrile:water (25:75, v-v) and filtered through a 0.22-μm filter for LC-MS analysis.

Plant hormones were analyzed using LC-MS/MS using the UHPLC-Triple-Stage Quadrupole Mass Spectrometer (Thermo Scientific^TM^ Altis^TM^). Chromatographic separation was achieved on the Hypersil GOLD C_18_ Selectivity HPLC Columns (150 × 4.6 mm; 3 μm; Thermo Scientific^TM^) with mobile phases consisting of water (A) and acetonitrile (B), both containing 0.1 % formic acid, and the following linear gradient (flow rate, 0.5 mL/min): 0–10 min, 15 %–100 % B, followed by washing with 100 % B for 5 min and equilibration with 15 % B for 2 min. The injection volume was 10 μL, and the column temperature was maintained at 35 °C for each run. The MS parameters of Thermo ScientificTM Altis^TM^ were as follows: positive ion mode for IAA and negative mode for GA, ABA, SA, and JA, ion source of H-ESI, ion spray voltage of 3,000 V, sheath gas of 40 arbitrary units, aux gas of 15 arbitrary units, sweep gas of 0 arbitrary units, ion transfer tube gas temperature of 350 °C, vaporizer temperature of 350 °C, collision energy of 20 eV, CID gas of 2 mTorr, and full width at half maximum (FWHM) 0.4 Da of Q1/Q3 mass. The characteristic Multiple Reaction Monitoring (MRM) transitions (precursor ion → product ion) were 176.2 → 130 for IAA; 263.2 → 153.1, 263.3 → 204.1, 263.3 → 219.1 for ABA; 347.2 → 259.1, 347.2 → 273 for GA1; 345.1 → 143, 345.1 → 239 for GA3; 137.1 → 93.15, 137.1 → 65.1 for SA; 209.15 → 59.05, 209.15 → 93.04 for JA; 178.2 → 132 for D2-IAA; 269.2 → 159.1 for D6-ABA; 349.1 → 261.1 for D2-GA1; 141.0 → 97.0 for D4-SA; 211.0 → 61.0 for D2-JA.

### *Striga hermonthica* Seed Germination Bioassays.

*Striga* seed germination bioassay was carried out based on the protocol of refs. 48 and 51. Briefly, 10-d-old preconditioning *Striga* seeds were supplied with 50 μL of extracted root exudates of different rice genotypes. After application, *Striga* seeds were incubated at 30 °C in the dark for 24 h. Germinated (seeds with radicle) and nongerminated seeds were counted under a binocular microscope to calculate germination rate (%) by using SeedQuant software (52).

### RNA Library Preparation and Transcriptomic Analysis.

Total rice root RNA was extracted with TRIzol™ (Invitrogen, https://www.thermofisher.com/de/de/home.html) using a Direct-zol RNA Miniprep Plus Kit following the manufacturer’s instructions (ZYMO RESEARCH; USA). RNA quality was checked with an Agilent 2100 Bioanalyzer, and RNA concentration was measured using a Qubit 3.0 Fluorometer. The cDNA libraries were constructed following standard protocols and paired‐end sequenced on an Illumina NextSeq Sequencer (Illumina HiSeq 4000) by Novogene Bioinformatics Technology Co., Ltd. Total reads were mapped to the rice transcripts using HISAT2 (53). Differential gene expression was examined using DESeq2 and established by false discovery rate (FDR) ≤ 0.05 (54).

### Plant Material and Growth Conditions for *R. irregularis* Root Colonization.

Seed of wild type (cultivar Nipponbare) and independent lines of four *Osmax1*-rice mutants (*Os900, Os900/1400-4BI, Os1400-12SIII and -12SIV)* were germinated in pots containing sand and incubated for 10 d in a growth chamber under a 14-h light (23 °C)/10-h dark (21 °C). Plants were inoculated with ~1,000 sterile spores of *Rhizophagus irregularis* DAOM 197198 (Agronutrition, Labège, France). Plants were grown in sterile quartz sand in a growth chamber with the same regime described before and watered with a modified Long-Ashton (LA) solution containing 3.2 μM Na_2_HPO_4_·12H_2_O.

Mycorrhizal roots were collected at two time points: 10 d post inoculation (dpi) and 40 dpi corresponding to the early and later stages of the mycorrhization process. For the molecular analyses, roots were immediately frozen in liquid nitrogen and stored at −80 °C. At the last time point (40 dpi), mycorrhizal roots were stained with cotton blue (0.1% in lactic acid), and the mycorrhizal colonization level was determined according to Trouvelot et al. (55).

### Transcript Analysis of Mycorrhizal Plants.

Total RNA was extracted from rice roots using the Qiagen Plant RNeasy Kit according to the manufacturer’s instructions (Qiagen, Hilden; Germany). Following the producer’s instructions, samples were treated with TURBO™ DNase (ThermoFisher). The RNA samples were routinely checked for DNA contamination through PCR analysis. Single-strand cDNA was synthesized from 1 μg of total RNA using Super-Script II (Invitrogen), according to the instructions in the user manual. Quantitative RT-PCR (qRT-PCR) was performed using a Rotor-Gene Q 5plex HRM Platform (Qiagen). Each reaction was carried out in a total volume of 15 μL containing 2 μL of diluted cDNA (about 10 ng), 7.5 μL of 2 × SYBR Green Reaction Mix, and 2.75 μL of each primer (3 μM). The following PCR program was used: 95 °C for 90 s, 40 cycles of 95 °C for 15 s, and 60 °C for 30 s. A melting curve (80 steps with a heating rate of 0.5 °C per 10 s and a continuous fluorescence measurement) was recorded at the end of each run to exclude the generation of nonspecific PCR products.

All reactions were performed on at least three biological and two technical replicates. Baseline range and take-off values were automatically calculated using Rotor-Gene Q 5plex software.

The transcript level of *OsPt11* (an AM marker gene) was normalized using the *OsRubQ1* housekeeping gene (41). Only take-off values leading to a mean with a standard deviation below 0.5 were considered. Statistical elaborations were performed using PAST statistical (version 4) (56).

### Ethynyl Deoxyuridine (EdU) Staining for Cell Proliferation Analysis.

For the EdU staining, the seedlings were transferred to 50 mL falcon tubes containing 2 μM 5-ethynyl-2′-deoxyuridine (EdU) in dH_2_O, so that the roots were completely submerged in the solution, and kept there for 2 h. The EdU staining was performed as described previously, using the Click-iT EdU Alexa Fluor 647 Imaging Kit (Invitrogen, ThermoFisher Scientific, USA) (57). The roots were cleared in CLEARSEE clearing solution (58) at 4 °C in darkness for 2 wk, and cell walls were counterstained with 0.1 % Calcofluor White M2R in CLEARSEE overnight in darkness. After washing the roots in CLEARSEE, they were imaged using an inverted confocal microscope (LSM 710, Zeiss) and a 20× objective. Calcofluor White was excited with 405 nm and detected in a detection range of 410–585 nm. Alexa 647 was excited with 633 nm and detected in a detection range of 638–755 nm.

### Statistical Analysis.

Data are represented as mean and their variations as SD. The statistical significance was determined by the two tailed unpaired Student *t* test or one-way ANOVA and Tukey’s multiple comparison test, using a probability level of *P* < 0.05. All statistical elaborations were performed using GraphPad Prism 9.

## Supplementary Material

Appendix 01 (PDF)

Dataset S01 (XLS)

Dataset S02 (XLSX)

## Data Availability

Anonymized RNAseq data have been deposited in NCBI’s Gene Expression Omnibus (GEO) (GSE221837) (59). All other data are included in the article and/or supporting information.
