# Supplementary material for: Disruption of the rice 4-DEOXYOROBANCHOL HYDROXYLASE unravels specific functions of canonical strigolactones
Source: Proc Natl Acad Sci U S A. 2023 Oct 11;120(42):e2306263120. doi: 10.1073/pnas.2306263120 (PMC10589652; doi:10.1073/pnas.2306263120)
Supplement: Supplementary file 1 — Appendix 01 (PDF) [file pnas.2306263120.sapp.pdf]

PNAS

Supporting Information for

Disruption of the rice *4-DEOXYOROBANCHOL HYDROXYLASE* unravels specific functions of canonical strigolactones

Guan-Ting Erica Chen, Jian You Wang, Cristina Votta, Justine Braguy, Muhammad Jamil, Gwendolyn K Kirschner, Valentina Fiorilli, Lamis Berqdar, Aparna Balakrishna, Ikram Blilou, Luisa Lanfranco, and Salim Al-Babili

*Correspondence: Salim Al-Babili

Email: salim.babili@kaust.edu.sa

This PDF file includes:

Figures S1 to S17

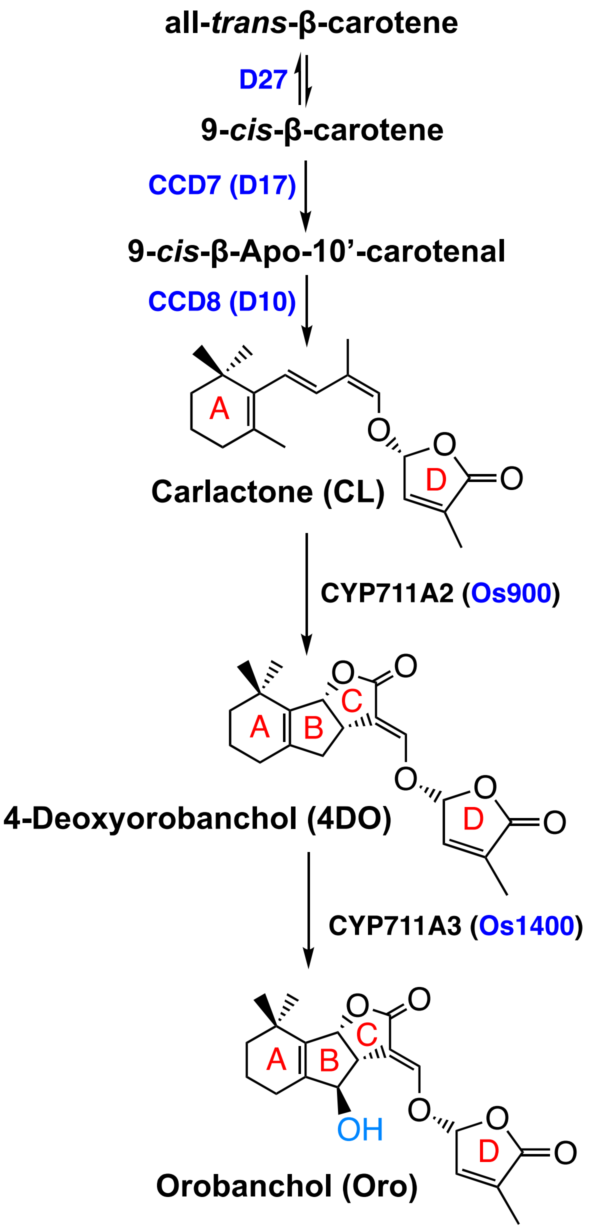

Fig. S1. Scheme of the biosynthesis of the rice canonical SLs. Canonical SL biosynthesis in rice starts with the reversible isomerization of all-*trans*- into 9-*cis*-β-carotene, catalyzed by the isomerase DWARF27 (D27). This step is followed by cleavage and rearrangement reactions mediated by the CAROTENOID CLEAVAGE DIOXYGENASE 7 (CCD7; D17 in rice) and CCD8 (D10 in rice), which yield carlactone (CL). The cytochrome P450 CYP711A2 (Os900) enzyme forms 4-deoxyorobanchol (4DO) from CL through repeated oxygenation and ring closure, while the homologous enzyme CYP711A3 (Os1400) hydroxylates 4DO into Orobanchol (Oro). The detailed SL biosynthesis pathway depicted in Fig.1C.

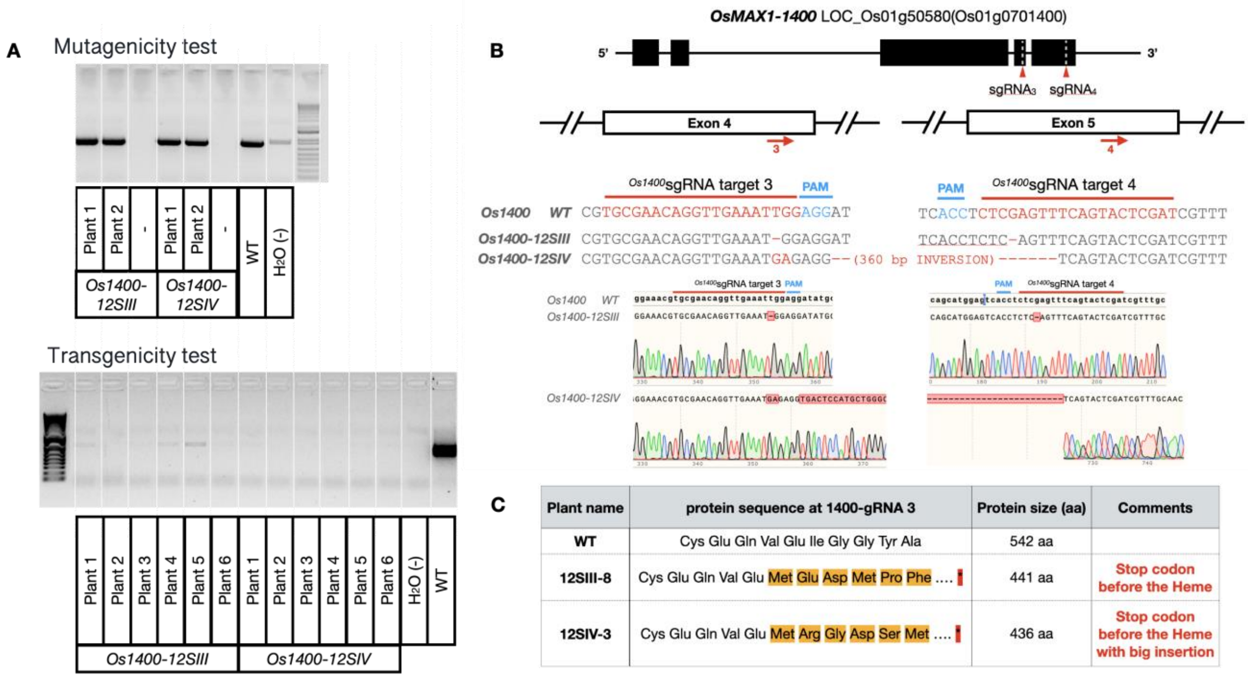

Fig. S2. Genotyping of *Os1400*-KO lines. (**A**) Genomic DNA amplification of the region surrounding sgRNA target site in wild-type (WT) and *Os1400*-KO lines -*12SIII* and -*12SIV* (2 plants each) (up, mutagenicity test) and pRGEB32 region containing the two *Os1400* sgRNAs sequences (6 plants each) (down, transgenicity test). Water (H_2O) and the pRGEB32 vector containing the two *Os1400* sgRNAs sequences were used as a negative (-) and positive (+) control, respectively. (**B**) Sequencing details of two representative plants of the homozygous *Os1400*-KO lines showing the different mutations present in each line, aligned to the WT sequence for both *Os1400* sgRNAs target sites. (**C**) Prediction of protein sequences revealed an early stop codon before the heme-iron ligand signature that is necessary for P450 protein activity. Abbreviations: WT, wild-type.

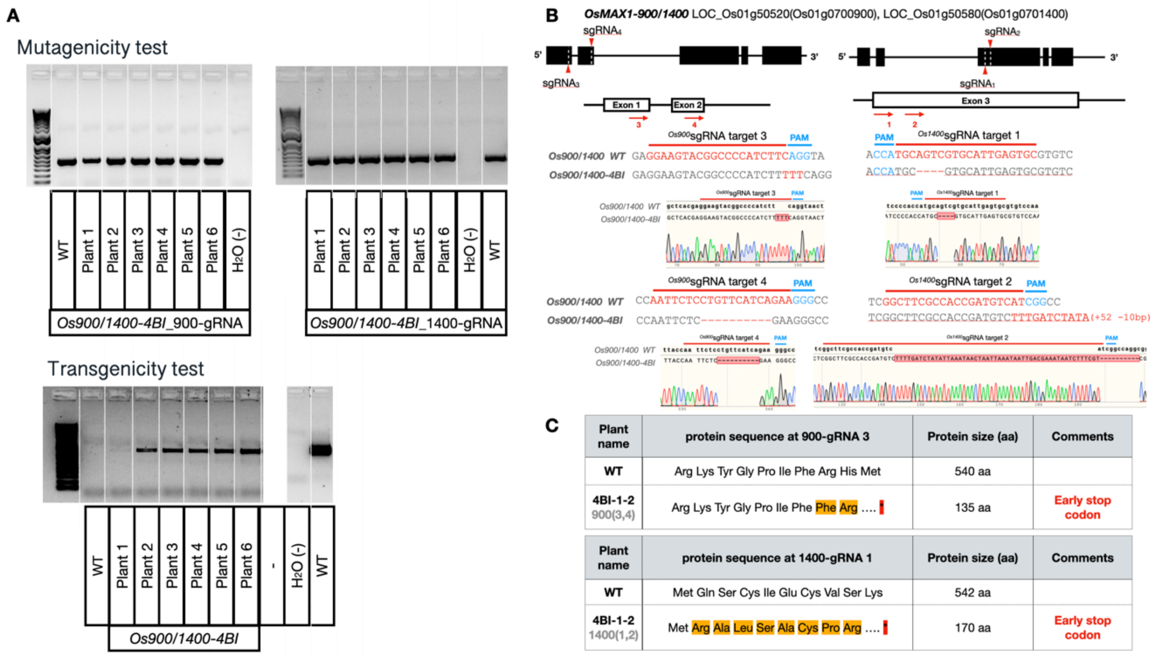

C

Plant name	protein sequence at 900-gRNA 3	Protein size (aa)	Comments
WT	Arg Lys Tyr Gly Pro Ile Phe Arg His Met	540 aa	
4BI-1-2 900(3,4)	Arg Lys Tyr Gly Pro Ile Phe Phe Arg	135 aa	Early stop codon

Plant name	protein sequence at 1400-gRNA 1	Protein size (aa)	Comments
WT	Met Gln Ser Cys Ile Glu Cys Val Ser Lys	542 aa	
4BI-1-2 1400(1,2)	Met Arg Ala Leu Ser Ala Cys Pro Arg	170 aa	Early stop codon

Fig. S3. Genotyping of *Os900/1400*-KO lines. (**A**) Genomic DNA amplification of the region surrounding sgRNA target site in wild-type (WT) and *Os900/1400*-KO line *4BI* (6 plants) (up, mutagenicity test) and pRGEB32 region containing the two *Os900* sgRNA and *Os1400* sgRNA sequences (down, transgenicity test). Water (H_2O) and the pRGEB32 vector containing the two *Os900* sgRNA and *Os1400* sgRNA sequences were used as a negative (-) and positive (+) control, respectively. (**B**) Sequencing details of two representative plants of the homozygous *Os900/1400*-KO line showing the different mutations present in each line, aligned to the WT sequence for both *Os900* sgRNA and *Os1400* sgRNA target sites. (**C**) Prediction of protein sequences revealed a early stop codon before the heme-iron ligand signature that is necessary for P450 protein activity. Abbreviations: WT, wild-type.

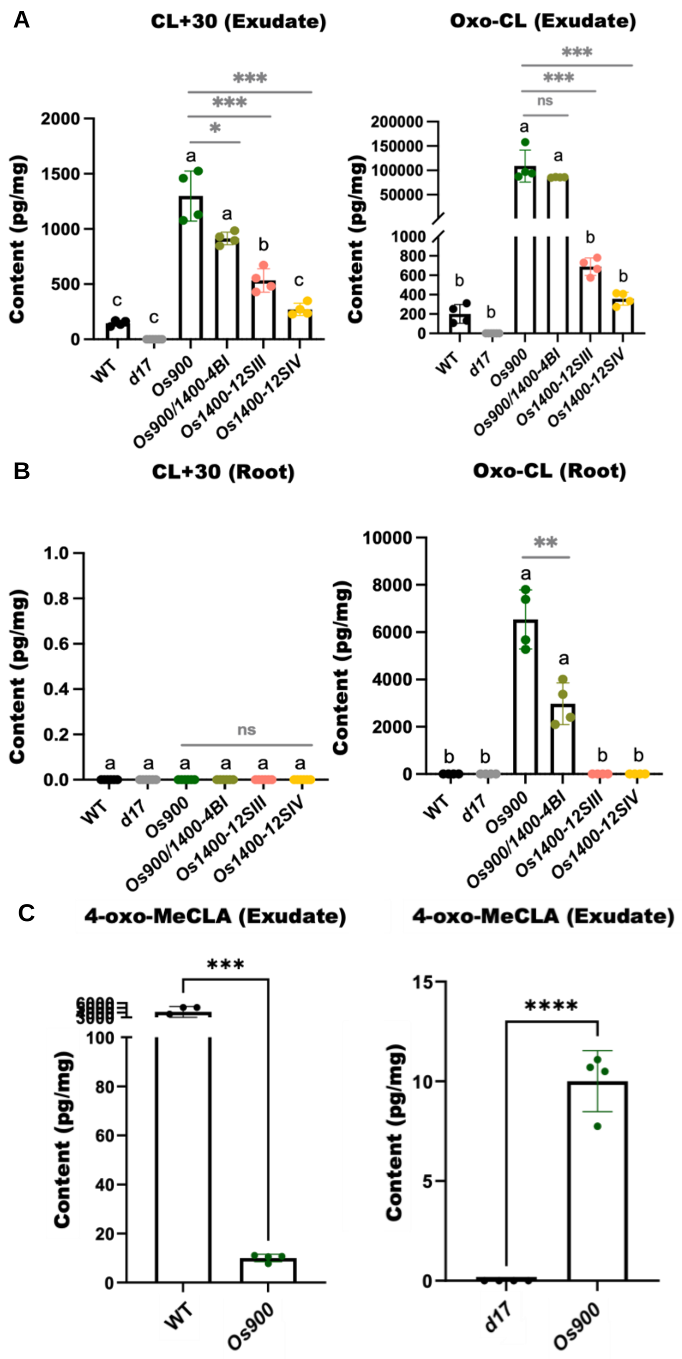

Fig. S4. Quantification of putative non-canonical SLs in WT, *Os900*-KO, *Os900/1400*-KO, *Os1400*-KO, and *d17* mutant lines. LC-MS quantification of SLs in the root exudates and root tissues of WT, *Os900*-KO line, *Os900/1400*-KO line, *Os1400*-KO lines, and *d17* mutant plants grown under low Pi conditions. Quantification of non-canonical SLs, CL+30 and Oxo-CL, in (**A**) root exudates and (**B**) root tissues of WT, *Os900*-KO line, *Os900/1400*-KO line, *Os1400*-KO lines, and *d17* mutant plants grown under constant low-Pi conditions. (**C**) Quantification of 4-oxo-MeCLA (indicated in Fig. 1A) in root exudates of WT, *Os900*-KO, and *d17* plants. 4-oxo-MeCLA was present in *Os900*-KO exudate at a low level (less than 4% of that of the WT), but not detectable in the *d17* exudates. The data are presented as means ± SD of 4 biological replicates. Asterisks indicate statistically significant differences as compared to control by two tailed unpaired Student *t* test (**P* < 0.05, ***P* < 0.01; ****P* < 0.001; *****P* < 0.0001). Abbreviations: CL, carlactone; 4-oxo-MeCLA, Methyl 4-oxo-carlactonoate; WT, wild-type; ns, non-significant.

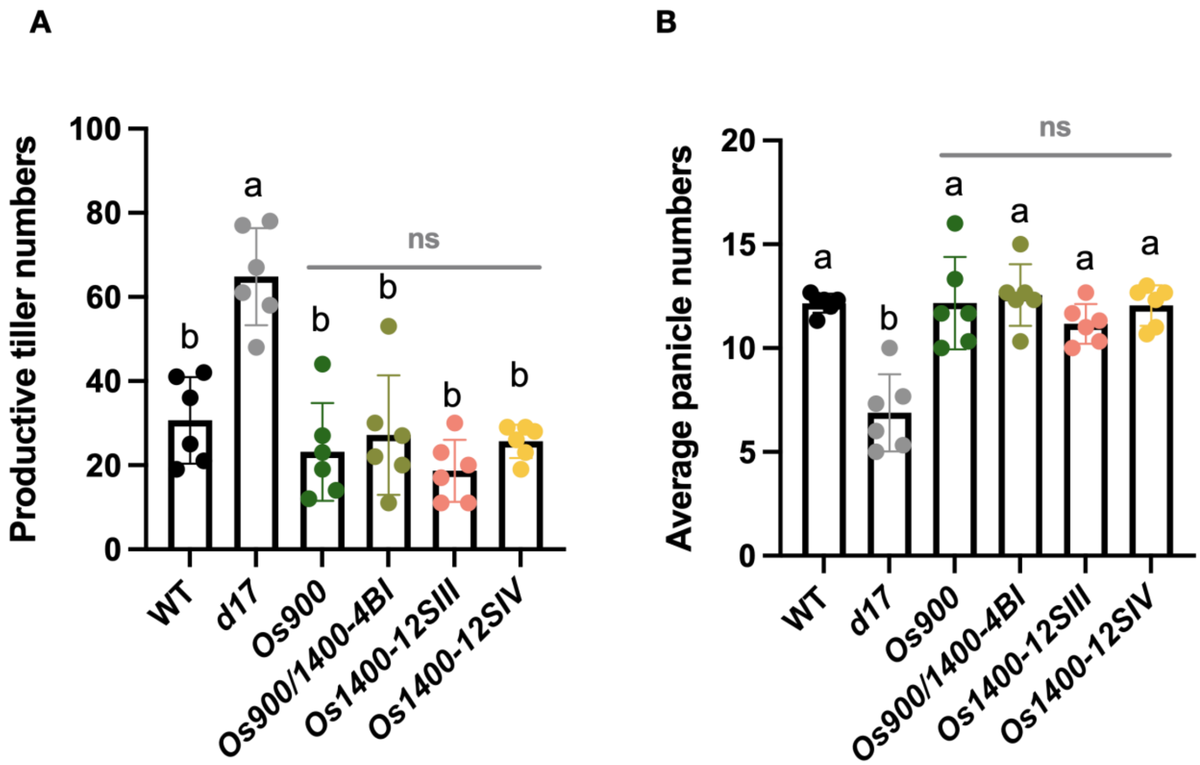

Fig S5. Phenotypic characterization of WT, *Os900*-KO, *Os900/1400*-KO, *Os1400*-KO, and *d17* mutant plants grown in soil. (**A**) Number of productive tillers, and (**B**) average panicle numbers. The data are presented as means ± SD of 6 biological replicates. Significant values determined by one-way ANOVA are shown with different letter ($P < 0.05$) when compared to WT, and asterisks indicate statistically significant differences as compared to control by two tailed unpaired Student *t* test (*$P < 0.05$, **$P < 0.01$; *******$P < 0.001$; ****$P < 0.0001$). Abbreviations: WT, wild-type; ns, non-significant.

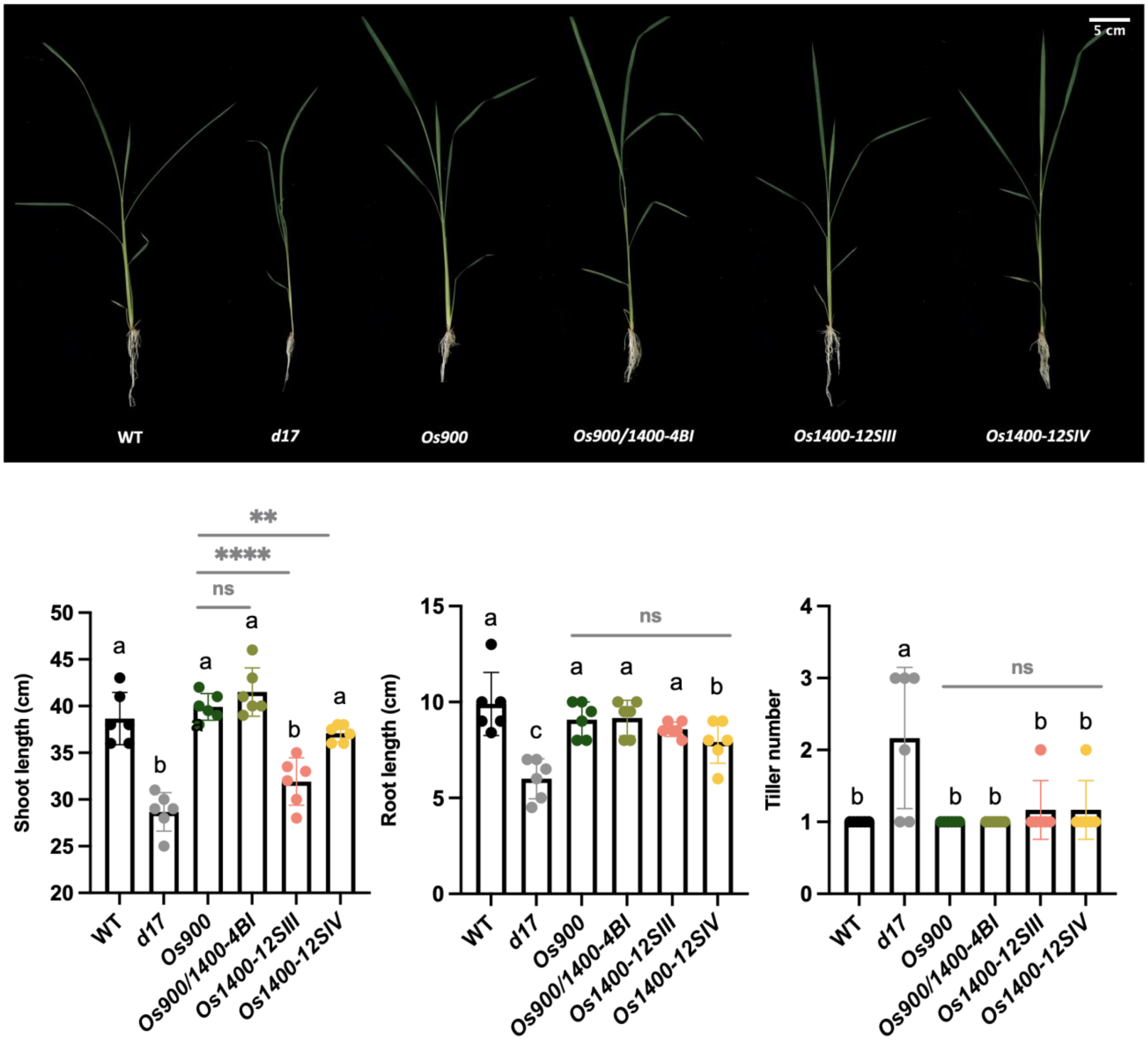

Fig. S6. Shoot and root phenotypes of WT, *Os900*-KO, *Os900/1400*-KO, *Os1400*-KO, and *d17* mutants grown hydroponically under normal (+Pi) conditions. The data are presented as means ± SD of 6 biological replicates. Significant values determined by one-way ANOVA are shown with different letter ($P < 0.05$) when compared to WT, and asterisks indicate statistically significant differences as compared to control by two tailed unpaired Student *t* test (*$P < 0.05$, **$P < 0.01$; ***$P < 0.001$; ****$P < 0.0001$). Scale bar, 5 cm. Abbreviations: WT, wild-type; ns, non-significant.

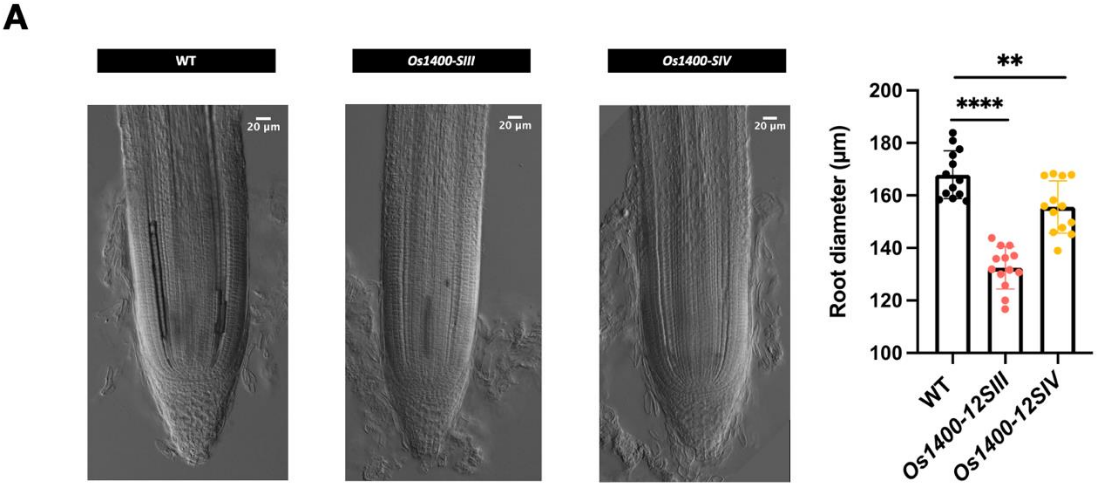

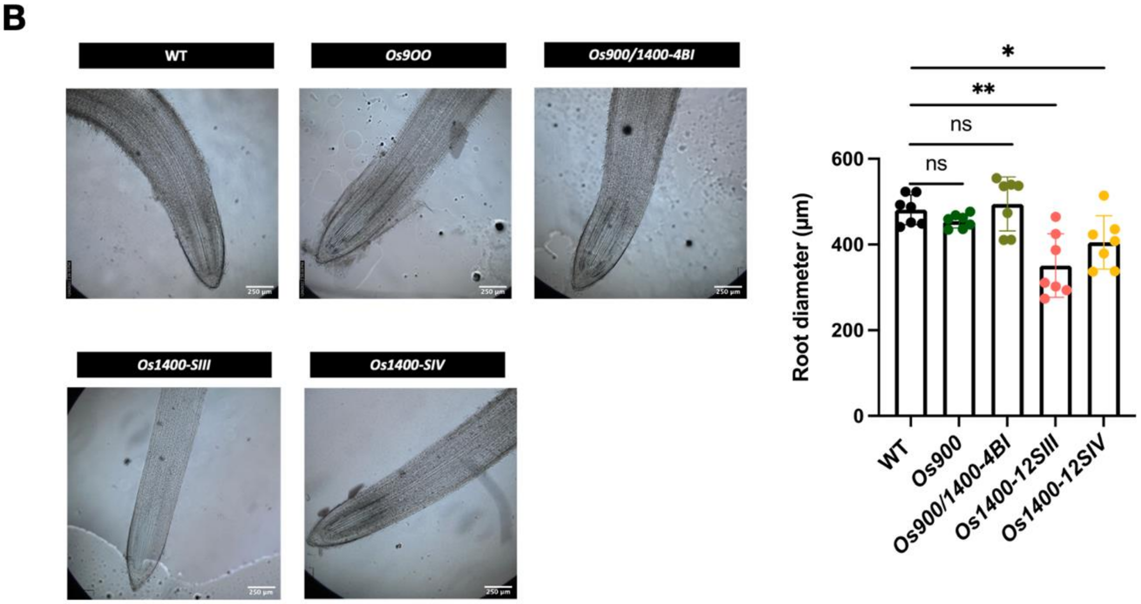

Fig. S7. Root width (diameter) measurement. (A) Root diameter of 2-week-old rice seedlings of Nipponbare WT, and *Os1400*-KO lines grown under normal (+Pi) hydroponic condition. **(B)** Roots picture of 3-week-old of Nipponbare WT, *Os900*-KO, *Os900/1400*-KO, and *Os1400*-KO seedlings grown under normal (+Pi) hydroponic condition. The data are presented as means ± SD of 7 biological replicates for (A), 13 for (B). Significant values determined by one-way ANOVA are shown with different letter ($P < 0.05$) when compared to WT, and asterisks indicate statistically significant differences as compared to control by two tailed unpaired Student *t* test (*$P < 0.05$, **$P < 0.01$; ***$P < 0.001$; ****$P < 0.0001$). Scale bar, 250 µm. Abbreviations: WT, wild-type; ns, non-significant.

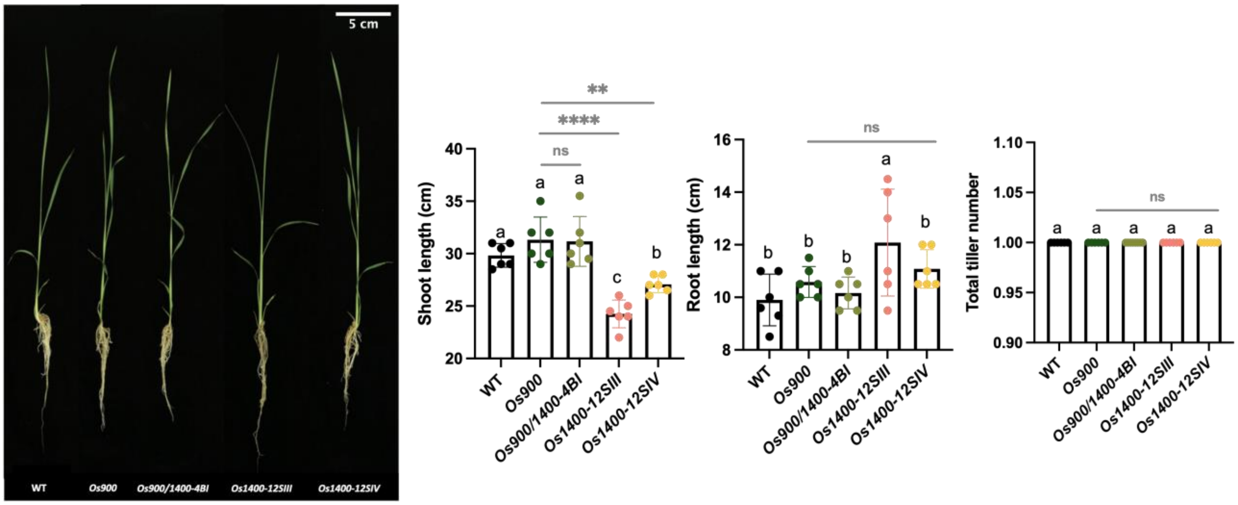

Fig. S8. Shoot and root phenotypes of WT, *Os900*-KO, *Os900/1400*-KO, *Os1400*-KO, and *d17* mutants grown hydroponically under phosphate deficient (low-Pi) conditions. The data are presented as means ± SD of 6 biological replicates. Significant values determined by one-way ANOVA are shown with different letter ($P < 0.05$) when compared to WT, and asterisks indicate statistically significant differences as compared to control by two tailed unpaired Student *t* test (*$P < 0.05$, ** $P < 0.01$; *** $P < 0.001$; **** $P < 0.0001$). Scale bar, 5 cm. Abbreviations: WT, wild-type; ns, non-significant.

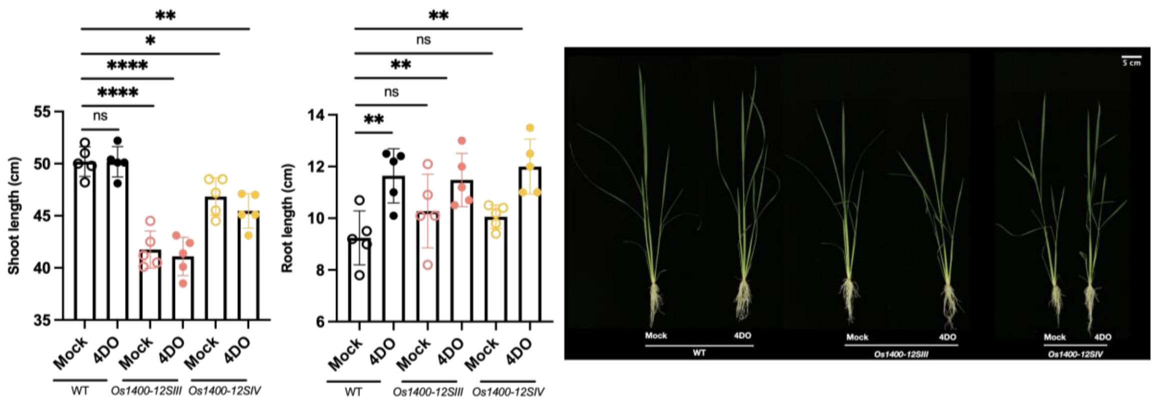

Fig. S9. Effects of 4DO application at 300 nM concentration on rice growth under normal (+Pi) hydroponic condition. Shoot and root phenotypes of WT and *Os1400*-KO lines grown hydroponically in the presence and absence (Mock) of 4DO (at 300 nM concentration). The data are presented as means ± SD of 5 biological replicates. Asterisks indicate statistically significant differences as compared to control by two tailed unpaired Student *t* test (*$P < 0.05$, ** $P < 0.01$; ***$P < 0.001$; ****$P < 0.0001$). Scale bar, 5 cm. Abbreviation: 4DO, 4-deoxyorobanchol; WT, wild-type; ns, non-significant.

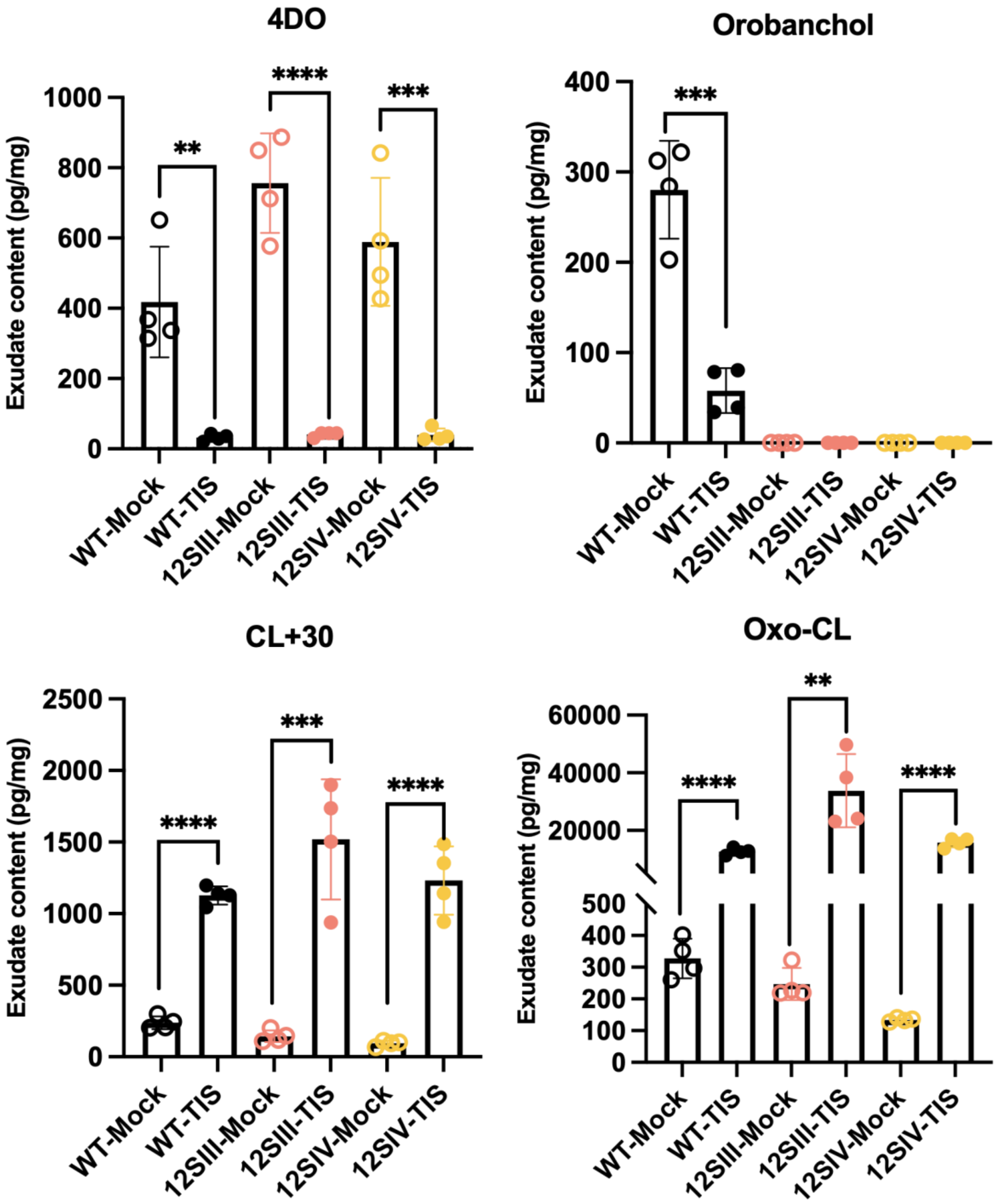

Fig. S10. Effect of TIS08 treatment on the amount of different rice SLs in root exudates of WT and *Os1400*-KO rice mutants. The data are presented as means of 4 biological replicates. Asterisks indicate statistically significant differences as compared to control (Mock) within each line by two tailed unpaired Student *t* test (**P* < 0.05, ***P* < 0.01; ****P* < 0.001; *****P* < 0.0001). Abbreviations: WT, wild-type; TIS, TIS108; ns, non-significant.

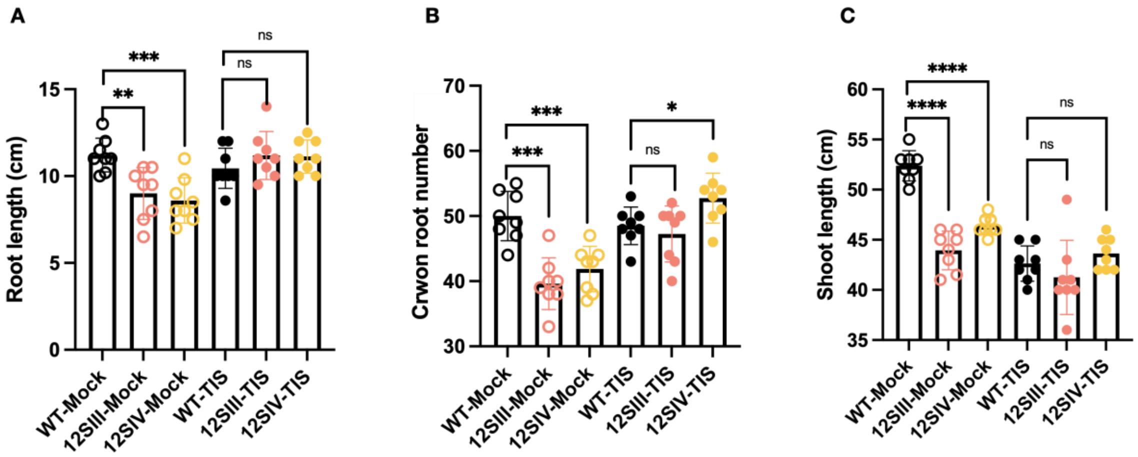

Fig. S11. Effect of TIS108 treatment on rice growth under normal (+Pi) hydroponic condition. (**A**) Root length, (**B**) number of crown roots, and (**C**) shoot length. The data are presented as means ± SD of ≥7 biological replicates. Significant values determined by one-way ANOVA are shown with different letter (*P* < 0.05) when compared to WT, and asterisks indicate statistically significant differences as compared to control by two tailed unpaired Student *t* test (**P* < 0.05, ***P* < 0.01; ****P* < 0.001; *****P* < 0.0001). Abbreviations: WT, wild-type; TIS, TIS108; ns, non-significant.

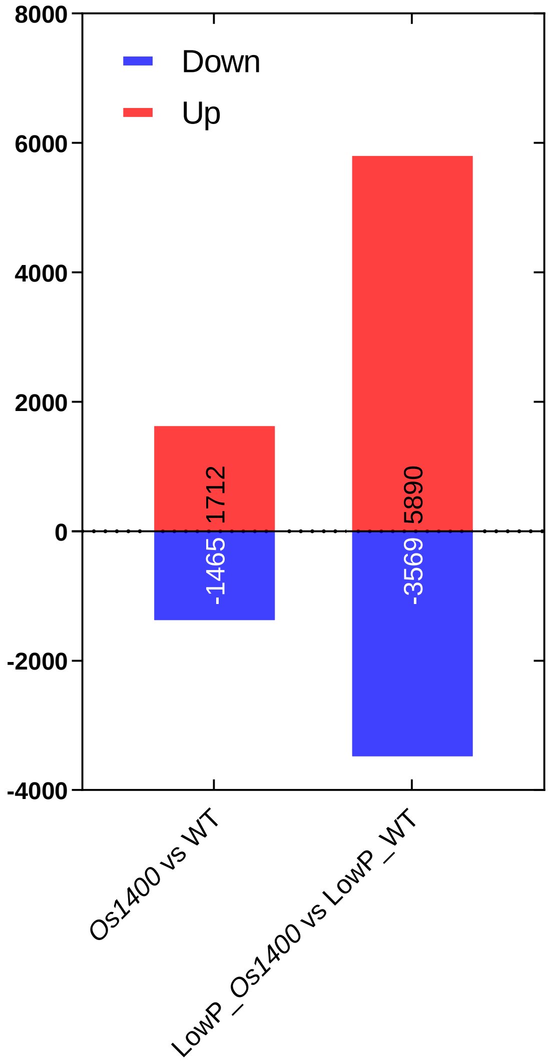

Fig. S12. Differentially expressed genes (DEGs) under normal (+Pi) and phosphate deficient (low-Pi) conditions. Numbers of the significantly expressed genes (FDR < 0.05). Up- and down-regulated genes are shown in red and blue bars, respectively. Abbreviations: WT, wild-type.

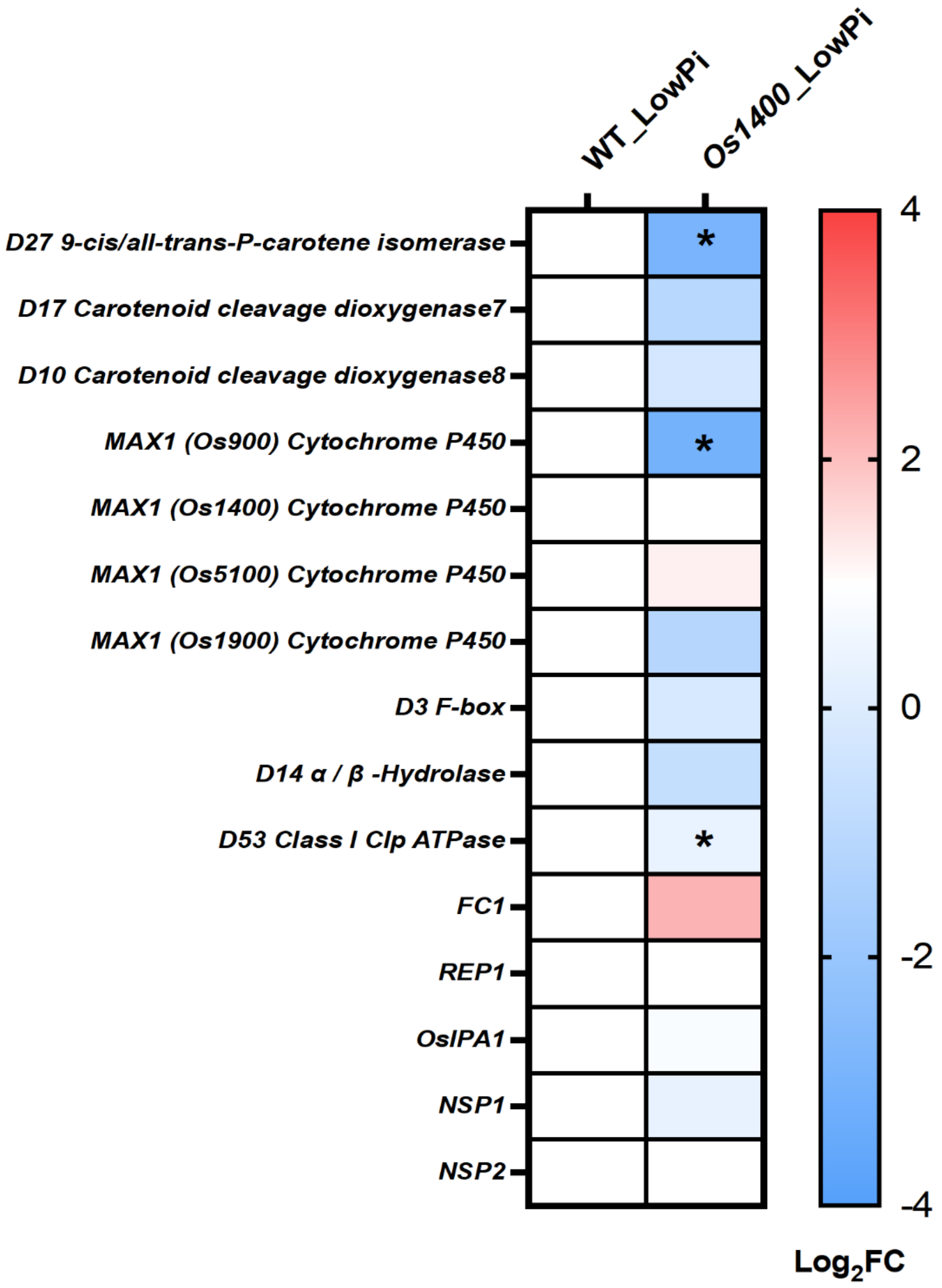

Fig. S13. Differentially expressed genes (DEGs) related to SL biosynthesis and signaling. Expression pattern is shown in log_2FoldChange (Log_2FC). Statistically significant differences are indicated by adjusted *P*-value (**P* < 0.05).

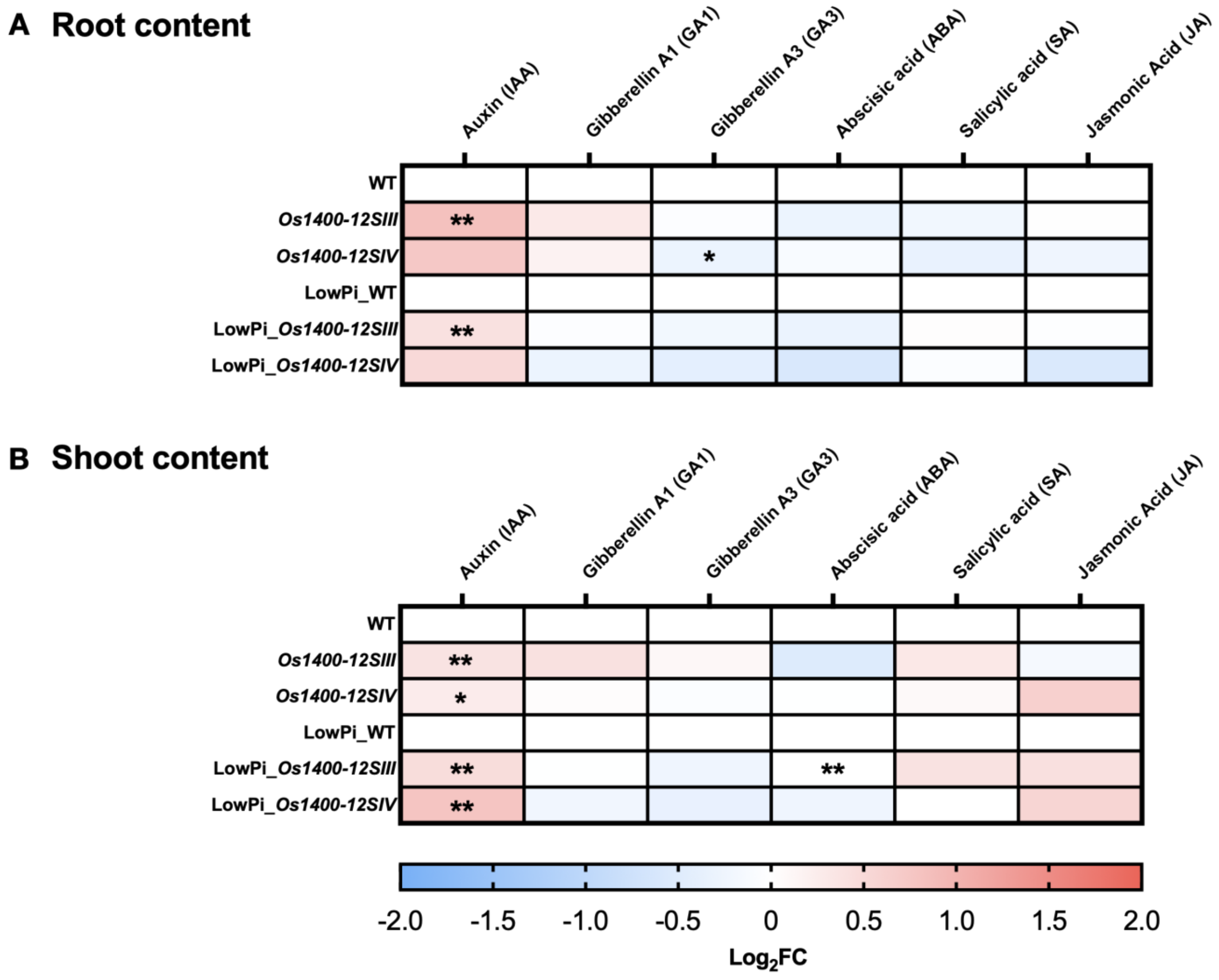

Fig. S14. Hormone profile under normal (+Pi) and phosphate deficient (low-Pi) conditions. Heatmap showing relative content of analyzed hormones in (**A**) roots and (**B**) shoot bases (root-shoot junction), in comparison to WT. For each hormone, the corresponding WT value was set to 1. Asterisks indicate statistically significant differences as compared to control by two tailed unpaired Student *t* test (**P* < 0.05, ***P* < 0.01). Abbreviation: WT, wild-type.

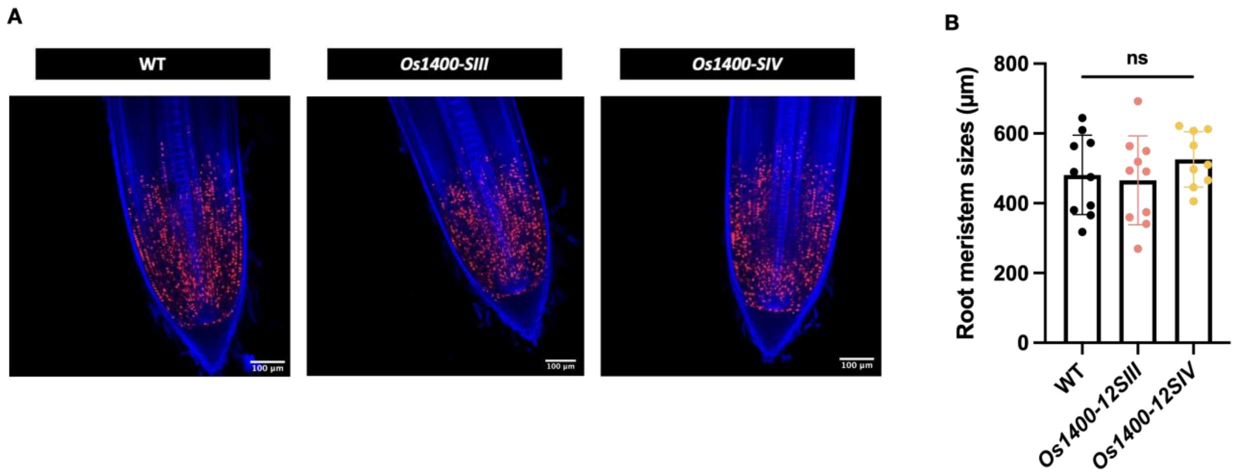

Fig. S15. Characterization of root development of *Os1400*-KO lines at cellular level.
(**A** and **B**) Ethynyl deoxyuridine (EdU) staining for cell proliferation analysis. Confocal images of rice roots showing dividing cells as captured by EdU staining in Zeiss LSM 710 inverted confocal microscope. Root meristem length of 10-day-old rice seedlings of WT and *Os1400-KO* lines. Dividing EdU-stained nuclei are shown in red; cell walls counterstained with 0.1 % Calcofluor White M2R are shown in blue. Images were acquired using the tile scan function in the Zen software with automatized stitching. Regions of interest were divided into multiple tiles and imaged individually. The tiles were then combined via automatic stitching to create a large overview image. Images are representative of the total number (n ≥ 9) of seedlings that were studied. The data are all presented as means ± SD of 6 biological replicates. Significant values determined by one-way ANOVA are shown with different letter ($P < 0.05$) when compared to WT, and asterisks indicate statistically significant differences as compared to control by two tailed unpaired Student *t* test (*$P < 0.05$, **$P < 0.01$; ***$P < 0.001$; ****$P < 0.0001$). Scale bar, 100 μm. Abbreviations: WT, wild-type; ns, non-significant.

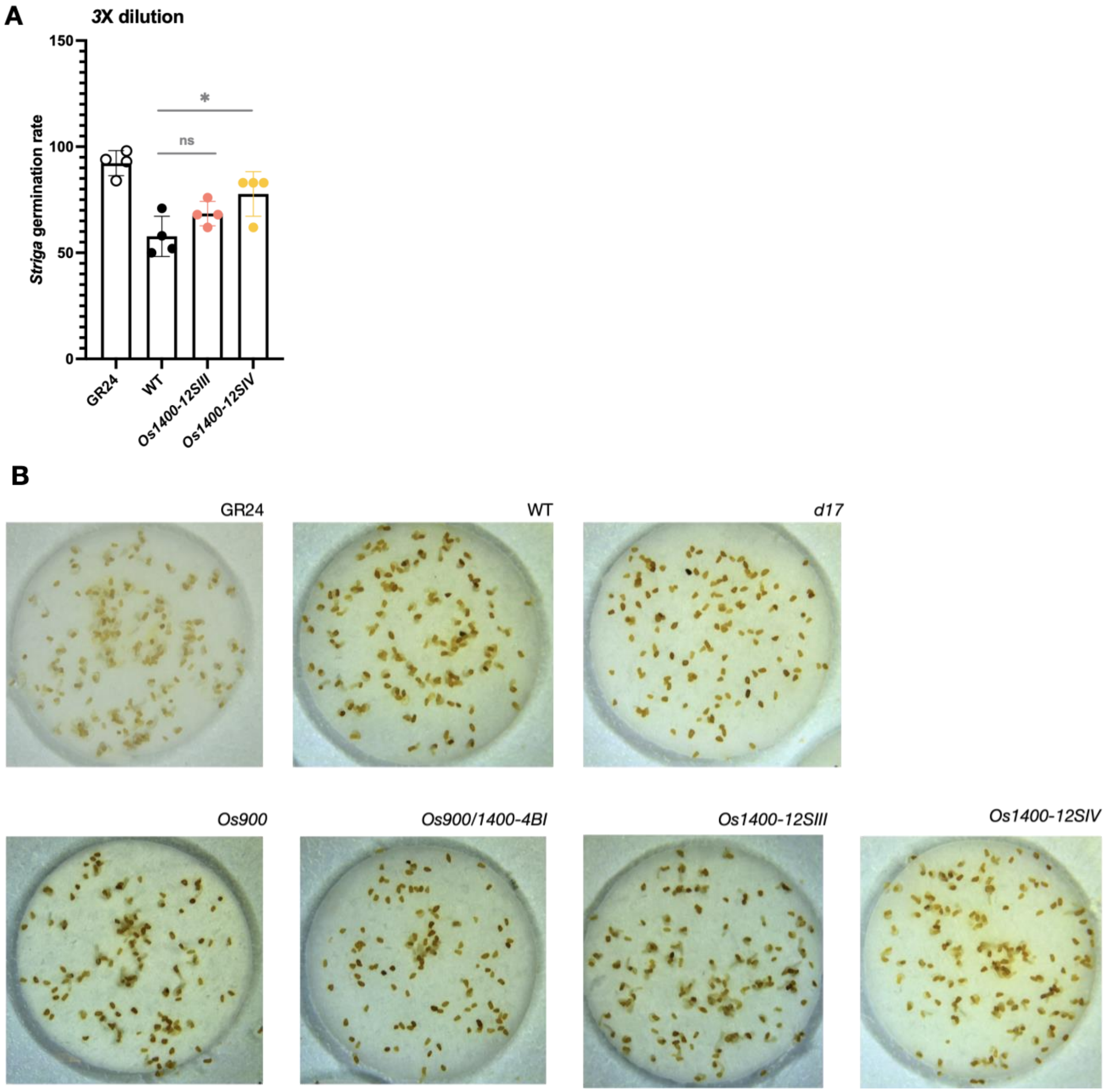

Fig. S16. *Striga* seed germination assays. (**A**) Seed Germination of *Striga hermonthica*, a root parasitic weed, upon treatment with 3X dilution and 1X dilution (**B**) of isolated root exudates. Significant values determined by two tailed unpaired Student *t* test (*$P < 0.05$, **$P < 0.01$; ***$P < 0.001$; ****$P < 0.0001$). Abbreviations: WT, wild-type; ns, non-significant.

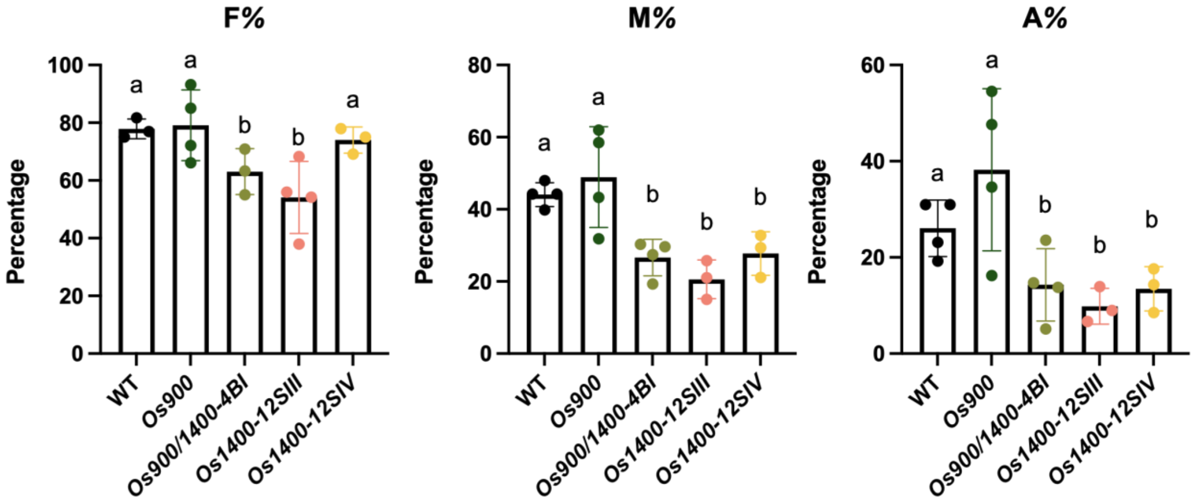

Fig. S17. Evaluation of AM colonization in *Osmax1* mutant lines. Mycorrhizal colonization of WT, *Os900/1400*-KO, and *Os1400*-KO lines by the AM fungus *Rhizophagus irregularis* at 40 dpi. Degree of colonization expressed as mycorrhizal frequency (F %), intensity (M %), and arbuscule abundance (A %) in the root system of WT and *Osmax1* lines. The data are presented as means ± SD of 4 biological replicates for (A and B), and n≥3 for (C). Significant values determined by one-way ANOVA are shown with different letter ($P < 0.05$) when compared to WT. Abbreviations: WT, wild-type; dpi, days post inoculation.

Table S1 (separate file) DEGs under normal condition.

Table S2 (separate file) DEGs under low-Pi condition.

Table S3 (separate file) Primer sequences used in this study.

Dataset S1 (separate file). Rice transcriptome of *Os1400* mutants under normal and low-Pi conditions.
